# Fine-grained statistical structure of speech

**DOI:** 10.1371/journal.pone.0230233

**Published:** 2020-03-20

**Authors:** François Deloche

**Affiliations:** Centre d’analyse et de mathématique sociales, CNRS, EHESS, Paris, France; Universidad de Salamanca, SPAIN

## Abstract

In spite of its acoustic diversity, the speech signal presents statistical regularities that can be exploited by biological or artificial systems for efficient coding. Independent Component Analysis (ICA) revealed that on small time scales (∼ 10 ms), the overall structure of speech is well captured by a time-frequency representation whose frequency selectivity follows the same power law in the high frequency range 1–8 kHz as cochlear frequency selectivity in mammals. Variations in the power-law exponent, i.e. different time-frequency trade-offs, have been shown to provide additional adaptation to phonetic categories. Here, we adopt a parametric approach to investigate the variations of the exponent at a finer level of speech. The estimation procedure is based on a measure that reflects the sparsity of decompositions in a set of Gabor dictionaries whose atoms are Gaussian-modulated sinusoids. We examine the variations of the exponent associated with the best decomposition, first at the level of phonemes, then at an intra-phonemic level. We show that this analysis offers a rich interpretation of the fine-grained statistical structure of speech, and that the exponent values can be related to key acoustic properties. Two main results are: i) for plosives, the exponent is lowered by the release bursts, concealing higher values during the opening phases; ii) for vowels, the exponent is bound to formant bandwidths and decreases with the degree of acoustic radiation at the lips. This work further suggests that an efficient coding strategy is to reduce frequency selectivity with sound intensity level, congruent with the nonlinear behavior of cochlear filtering.

## Introduction

Persons with normal hearing can effortlessly grasp the meaning of utterances from an acoustic signal that has a rich structure even on short time scales. Discovering the attributes that efficient speech processing systems need to incorporate can help us to better understand how hearing works. It is also important for the development of more effective hearing aids or automatic speech recognition devices. A concern in the study of these systems is how they can efficiently represent speech sounds, a question that can be addressed within the framework of Shannon’s information theory. The assumption that the brain processes sensory signals by optimizing an information theoretic criterion is called the efficient coding hypothesis [1]. The original formulation of this hypothesis states that sensory systems reduce the redundancy of neural signals in order to make maximum use of coding capacity [2–4]. Redundancy is reduced if natural stimuli are represented with a set of features as independent as possible, a principle that is translated in practice into a method of data analysis known as Independent Component Analysis (ICA). ICA seeks a transformation—in most works linear—that makes the components of high dimensional data statistically independent. When applied to a specific class of data, the representation produced by ICA reveals its overall structure [5], and at the same time gives an insight into an efficient sensory coding scheme [6]. The sparseness of activation patterns is another popular criterion for coding efficiency [7]. It corresponds both to the idea that the brain seeks to limit neural activity in an attempt to save neuronal resources, and to a sparsity hypothesis on the underlying structure of the input signals. Sparse coding is similar to Independent Component Analysis when the independent components are associated with sparse activations, and algorithms of sparse coding have been used to investigate the structure of sensory signals as well [8, 9]. The efficient coding approach initiated numerous studies on the properties of the visual system [10], and is also the basis for comparable studies on the auditory system, whether on peripheral processing [9, 11, 12], or more recently on higher level processing (e.g. modulation filters) [13–16].

Independent Component Analysis applied to speech waveforms results in a time-frequency representation whose frequency selectivity follows the same power law in the high frequency range 1–8 kHz as the frequency selectivity of the mammalian cochlea [11]. While this finding is consistent with the hypothesis that speech statistics are adapted to peripheral auditory processing, it cannot be easily interpreted in terms of signal structure. The diversity of phones in a language makes it difficult to offer a single interpretation of the decomposition revealed by ICA that would apply to any speech sound. In addition, it is possible that some regularities that are not captured by ICA, applied to speech data as a whole, exist at a finer level. In order to get a description of the statistical structure of speech based on concrete properties of the signal, one approach is to split the speech data into categories of sounds that share common acoustic features. In 2013, Stilp and Lewicki applied ICA to phonetic categories (e.g. fricatives, stops, affricates, vowels) instead of speech as a whole [17]. They found that the trade-off between time and frequency resolution was different depending on the class used at the input of ICA and assumed that the time-frequency resolution is mostly explained by the transiency of sounds within a class. Rapid changes in time would make the optimal filters shift towards a time representation with poorer frequency selectivity. This view, however, does not fully explain why vowels result in a representation that is more localized in time than fricatives for example. We also do not know if the predefined broad phonetic categories that were used are the most relevant to signal structure. Phonemes and categories of phonemes have been extensively described by their acoustic properties, but a comprehensive account of how these properties affect the statistical structure of speech is still missing.

This article aims to provide a description of the fine-grained statistical structure of speech using a simplified representation model. Previous work on the efficient coding of speech has shown consistent properties for the representations learned. When ICA is applied to speech data, it produces a bank of filters that resemble Gabor filters—i.e. Gaussian modulated sinusoids [9, 11, 18], whose frequency selectivity is related to center frequency by a power law [11]. Recent analyses took advantage of this fact and used one parameter, the regression slope of the quality factor *Q*_10_ on center frequency *f*_*c*_—on a logarithmic scale—to discriminate between representations learned from different speech data [17, 19]. The *Q*_10_/*f*_*c*_ coefficient, referred to as the parameter *β* in this article, characterizes the time-frequency resolution trade-off in the high frequencies: frequency accuracy at the expense of time accuracy, or inversely. In this study, we fully adopt the parametric approach to analyze the statistical structure of speech at a fine subdivision of speech. We use a cost function that reflects the sparsity of decompositions in Gabor dictionaries to estimate *β*, then we investigate the variations of this single parameter over different phonetic categories or over different time points of single phonemes. The main acoustic properties that play a role in the determination of *β* can be inferred from these analyses. Along with actual speech data, we also use artificial signals with explicit structure to support the interpretation of the results. In addition to revealing the relationships between acoustic features and the best time-frequency trade-offs, this work further suggests that an efficient strategy for speech coding is to reduce frequency selectivity with sound intensity level, consistent with the nonlinear behavior of cochlear filtering.

## Materials and methods

The analyses in this work are based on the parameter *β*, the *Q*_10_ on *f*_*c*_ exponent, which has been shown to summarize many speech representations learned with Independent Component Analysis in previous work. The optimal *β* value is estimated for many subsets of speech data, using a set of overcomplete dictionaries of Gabor filters. The outline of the method is as follows: the speech slices are decomposed in the dictionaries indexed by *β*, and a cost function *h*(*β*) reflecting the lack of sparsity is computed for the resulting decompositions. The dictionary that minimizes the cost function averaged over the selected samples, offering the most sparse representation, provides an estimate of the best value *β*^⋆^ for the corresponding data:

$$\begin{array}{c} \beta^{⋆}=\text{arg}\;{\text{min}}_{\beta }\;h\left(\beta \right)\;. \end{array}$$

In the rest of the article, *β* refers to *β*^⋆^, the optimal choice of the parameter, when there is no possible confusion with the other values.

In the methods section, we first justify the representation model and describe the construction of the Gabor dictionaries. The cost function is then introduced, and at this occasion a formal link with ICA is proposed. The last subsection details the analyses that were conducted, both on real speech data and artificial signals.

### Gabor dictionaries and interpretations of the *β* parameter

The candidates for the best representations were a set of 30 overcomplete dictionaries of Gabor filters corresponding to different *β* values, ranging from 0.3 to 1.2 with a constant step. A Gabor filter *w*(*t*) is a Gaussian-modulated sinusoid (imaginary part is ignored), associated with the best time-frequency resolution allowed by the Heisenberg-Gabor inequality [20]. A more precise mathematical argument in favor of Gabor filters is that they offer the most sparse patterns for cross Wigner–Ville distributions [21]. This choice is also consistent with the filter shapes found empirically with ICA [9, 11, 18]. The equation of a Gabor filter is:

$$\begin{array}{c} w\left(t\right)=C\;\text{sin}\left(\omega t+\phi \right)\;\text{exp}\left(-\frac{{\left(t-\tau \right)}^{2}}{4\sigma_{t}^{2}}\right) \end{array}$$ (1)

where *τ*, *σ*_*t*_, *ϕ*, *f*_*c*_ are respectively the time shift, time deviation, phase, and center frequency, and *C* is a normalization factor that ensures that all atoms have the same root mean square value. Each dictionary was composed of 600 Gabor filters uniformly distributed in time, frequency and phase. The only parameter that remained to be set was the width of each atom, also determined by the frequency selectivity. A measure of frequency selectivity is the quality factor *Q*_10_, defined by *f*_*c*_ divided by the 10dB-bandwidth Δ*f*. When ICA is applied to sufficiently broad subclasses of speech sounds, the *Q*_10_ factor of the resulting filters, plotted against center frequency, is well fitted by a line on a *log-log* scale. The intercept was found redundant with the slope of the regression in recent analyses, with most of the lines crossing around the point (*f*_0_ = 1 kHz, *Q*_0_ = 2) for various speech data at the input of ICA [17, 19]. We relied on these previous studies by considering that the *β* parameter, corresponding to the regression slope of *Q*_10_ on *f*_*c*_, is a synthetic parameter of the representations learned.

**Fig 1:**
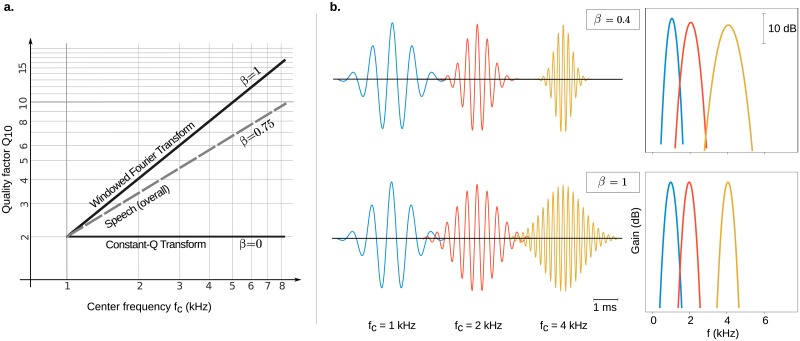
**a. Interpretation of the *β* parameter**. *β* is the slope of the quality factor *Q*_10_ on center frequency *f*_*c*_ (both axes are on a logarithmic scale). *β* = 1 characterizes unique resolution decompositions (e.g. Windowed Fourier Transform), whereas *β* = 0 characterizes multi-resolution decompositions (e.g. Constant-Q Transform, Wavelet Transform). The most sparse decomposition of speech is obtained with *β* = 0.75. **b. Examples of filters that compose the Gabor dictionaries indexed by *β***. These dictionaries are the candidates for the most sparse decompositions of the speech signals. The *β* parameter controls the time-frequency trade-off in the high frequencies: for *β* = 0.4, the filters are localized in time but poorly selective in frequency (top), contrarily to filters for the higher value *β* = 1 (bottom). **left**: time waveforms. **right**: Corresponding frequency responses (gain in dB).

The parameter *β* describes a fundamental property of time-frequency analysis as it makes the distinction between constant resolution (*β* = 1) and multi-resolution (*β* → 0) decompositions (Fig 1A). A constant resolution decomposition is associated with a unique characteristic bandwidth whereas in a multi-resolution decomposition, filter bandwidths are proportional to center frequencies. This parametrization corresponds to a class of dictionaries called flexible Gabor-wavelets or *α*–*atoms* in the field of time-frequency analysis, with the correspondence *β* = 1 − *α* [22]. Another interpretation of *β* is that it controls the time-frequency trade-off in the high frequency range (Fig 1B). We will say that the representation shifts towards a time decomposition when *β* approaches the minimum value.

The range for the *β* values [0.3, 1.2] was chosen to encompass all the values of *β* found in previous work. To ensure more diversity of filters, some randomness was added to the Q-factor with multiplicative noise. It follows that

$$\begin{array}{c} \text{log}\;Q_{10}\left(f\right)=\text{log}\;Q_{0}+\beta \left(\text{log}\;f-\text{log}\;f_{0}\right)+0.04\eta \end{array}$$ (2)

where the log is taken in base 10 and *η* is i.i.d. noise drawn from the normal distribution. As for the other parameters that follow a uniform distribution, the ranges were respectively [1–6.5 kHz], [2–14 ms] and [0, *π*] for center frequency, time shift and phase. The time shift did not cover the full range of the time window (T = 16 ms) in order to avoid potential boundary effects due to truncated filters.

### Data

The speech data was retrieved from the TIMIT database [23]. It provides recordings of sentences in American English as well as labels on their phonetic content segment by segment. Slices of 16 ms of speech were considered, representing *n* = 256 samples at *f*_*s*_ = 16 kHz. The slices were preprocessed with a filtering and normalization step. They were filtered by a high pass Butterworth filter of order 8 with a cut-off frequency at 1.5 kHz. The choice of a high cut-off frequency is not common for speech analysis as frequency components below *f*_0_ = 1 kHz also contain much information, but the focus of this study is on the high frequency region above the intersection point of the power laws at *f*_0_. In addition, the dictionaries are all the same at *f*_0_, meaning that the region of discrimination is in even higher frequencies. The normalization step was done by dividing each slice by its root mean square (RMS value). The TIMIT database indicates the time of releases for stops and affricates. This information was used several times in the analyses, in particular the closure part, which contains no high frequency information, was always ignored.

In addition to real speech data, we also analyzed artificial signals with similar structure to speech sounds. The generation of this synthetic data is described further in the paragraph Artificial signals.

### Cost function

Given some speech data, we need a criterion to select the best representation among the Gabor dictionaries. We adopted the view of sparse coding by considering that the sparseness of response activations has to be maximized [7]. We consider *n*–dimensional vectors *X*, representing the time waveforms of the preprocessed 16 ms slices. Our goal is to select the best set of filters *W*_*β*_ = (*W*_1_, …, *W*_*m*_) among the Gabor dictionaries indexed by *β*. Let $Y_{\beta }=W_{\beta }^{T}X$ be the output vectors obtained by decomposing the input vectors in the Gabor dictionaries. A raw measure of sparseness is expressed by the average sum of the response activations:

$$\begin{array}{c} h_{\text{raw}}\left(\beta \right)=E(\left|\right|Y_{\beta }{\left|\right|}_{1})=E(\sum_{i}|{\left[W_{\beta }^{T}X\right]}_{i}|)\;. \end{array}$$ (3)

This measure is the *L*_1_ norm of the output vector. This method differs slightly from many other sparse coding methods [8, 24] in that there is no attempt to *reconstruct* the input signal from the output. This corresponds to a first step of processing (an *analysis* step) where the complete set of decompositions is computed, but there is no subsequent selection of atoms.

The actual cost function included weights as normalization factors:

$$\begin{array}{c} h\left(\beta \right)=E(\sum_{i}\gamma_{i}|{\left[W_{\beta }^{T}X\right]}_{i}|)\;. \end{array}$$ (4)

The choice of the weights is explained in the paragraph Weighting strategy. In a final step, we normalized the cost function with *h*(*β*) set to 1 for the less sparse signals.

The cost defined as above measures the lack of structure, and related to a coarse measure of entropy, as explained in next paragraph. Average values of *h* over the set of Gabor dictionaries were considered simultaneously with *β*. Low values of *h* characterizes sounds that present structure, typically vowels. On the contrary, maximum values of *β* characterize sounds that are similar to noise (ex: obstruents: fricatives, stops…). Another interpretation of *h* that applies to many speech sounds is that it is a measure of localization. A signal with a single peak will be associated with a minimum cost *h*: *β* will reach the minimal value if this peak is on the time axis, or the maximal value if it is localized in frequency.

#### Link with Independent Component Analysis

The cost function has a formal link with the entropy minimization formulation of Independent Component Analysis [1, 5]. From this perspective, the goal of ICA is to minimize the mutual information between the components of the output vector *Y* = *W*^*T*^ *X*:

$$\begin{array}{c} I\left(Y_{i},\cdots ,Y_{m}\right)=\sum_{i}H\left(Y_{i}\right)-H\left(Y\right)\;. \end{array}$$ (5)

This expression is nonnegative and equals zero if and only if the components are independent. However, one needs a probabilistic model to estimate the sum of entropy terms. For speech, marginal distributions for various time-frequency decompositions are well approximated by Laplace distributions *q*(*y*) [25] characterized by:

$$\begin{array}{c} \text{log}[q(y)]=\text{log}\;\gamma /2-\gamma |y| \end{array}$$ (6)

where *γ* is a scale parameter proportional to the quadratic mean. The Laplace distribution encourages the sparsity of decomposition coefficients, as most of the values of *Y*_*i*_ are around zero under this prior. It has been used multiple times for ICA applied to speech [9, 17]. Under this prior, the entropy terms in the sum ∑_*i*_ *H*(*Y*_*i*_) can be replaced by cross-entropy terms, yielding the following approximation:

$$\begin{array}{c} \sum_{i}H\left(Y_{i}\right)\approx -E(\sum_{i}\text{log}\;q_{i}\left(y_{i}\right))=-\sum_{i}\text{log}\;\gamma_{i}/2+E(\sum_{i}\gamma_{i}|{\left[W_{\beta }^{T}X\right]}_{i}|)\;, \end{array}$$ (7)

where the variable term is the cost function previously defined (Eq 4).

The above reasoning did not take into account the last term −*H*(*Y*) in Eq 5. If *W* is a square matrix, this term is related to the entropy of the input and can be replaced by −log |det*W*|. This behaves as a penalty term that ensures that the column vectors of *W* represent all directions of the *n*-dimensional space and avoids the collapse of filters during learning. However, the penalty term has no natural expression for overcomplete families of filters (*m* > *n*) [26]. In our method, the diversity of filters is ensured by the construction of dictionaries, since the filters that compose the dictionaries are uniformly distributed in time, frequency, phase. Therefore, the dictionaries were considered to be on an equal footing and the penalty term was ignored. With this cost function, our method produced *β* values that are consistent with previous studies on the statistical structure of speech based on ICA, and some of the results in this work have been replicated using ICA as well (discussed further in Results and Discussion sections).

#### Weighting strategy

The actual cost function includes weights as normalization factors:

$$\begin{array}{c} h\left(\beta \right)=E(\sum_{i}\gamma \left(f_{i}\right)|{\left[W_{\beta }^{T}X\right]}_{i}|)\;. \end{array}$$ (8)

where *f*_*i*_ is the center frequency of the *i*-th filter and *γ*(*f*) typically is an increasing function of the frequency. We define three weighting strategies:

- *Strategy A* (raw scores): we make no difference between the components, setting *γ*(*f*) = *γ*_0_ to a constant.
- *Strategy B* (spectral whitening): we set *γ*(*f*) to be inversely proportional to the amplitude spectral density (for speech: +5dB/octave).
- *Strategy C* is a balance between the two strategies defined above. It consists in applying a slighter gain of +2.5dB/octave.

As seen in the previous paragraph, strategy B is mathematically justified and corresponds to the normalization of the marginal distributions. It ensures that medium frequencies do not override high frequencies due to the natural decrease in energy along the frequency axis. However, it has the opposite effect on high frequency sounds (e.g. sibilant fricatives), leading to erratic behaviors for these classes of sounds. The more naive Strategy A is also interesting in this respect because it considers the response patterns without any assumption about the global power spectrum. The results presented in the core of the article were obtained with the intermediary strategy (*Strategy C*). We also provide the central figure on phonemes, obtained with Strategies A&B, as supporting information (S1 Appendix).

### Data analysis

The results of two analyses on speech data are presented in this article. **Analysis 1** is an estimation of the *β* parameter for phonemes or categories of phonemes, in the continuation of Stilp and Lewicki’s work. **Analysis 2** goes further by describing the temporal behavior of *β* within phonemes.

**Fig 2:**
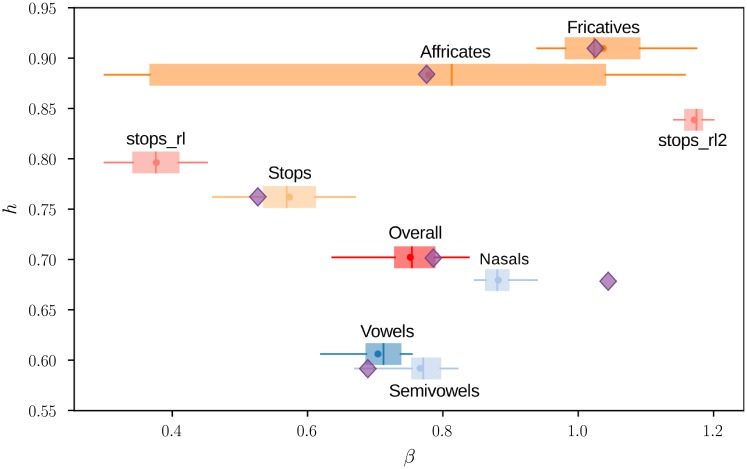
Distribution of American English broad phonetic categories in the (*β*, *h*) plane. The box plots show the quartiles Q1, Q2, Q3, the [5%, 95%] percentiles (whiskers) and the mean (dot) of the bootstrap distributions based on 800 samples for each category. *stops_rl* and *stops_rl2* are for first parts and second parts of stop releases (see paragraph Stops, fricatives, and affricates). The diamonds show the values found by Stilp and Lewicki for the same categories using Independent Component Analysis [17].

**Analysis 1**: The purpose of this analysis is to estimate the *β* value associated with the most sparse decomposition for different classes of speech sounds: broad phonetic categories (fricatives, stops, vowels…) or single phonemes. We retrieved occurrences from the TIMIT database for each class of speech sounds, randomly sampled from throughout the database: 400 occurrences for single phonemes, or 800 occurrences for phonetic categories. We used 800 samples for the phonetic categories to get more robust estimations of *β*^⋆^ since they contain a greater diversity of sounds. A 16 ms slice was selected at random for each occurrence, and the cost functions *h*(*β*) were computed according to Eq 8. The scores were then smoothed with a Gaussian filter (*σ* = 0.03) along the *β* axis, and the minimum score was obtained for *β* = *β*^⋆^. The values plotted in this article are the means and 70% confidence intervals of bootstrap distributions. They were obtained by repeating the estimation of *β*^⋆^ 3 000 times with re-sampled versions of the 400 (or 800) slices with repetitions. Alternatively, bootstrap distributions can be represented by box plots as done in Fig 2. Examples of histograms for the bootstrap distributions are shown in S2 Appendix. For the broad phonetic categories, the phonemes were divided into vowels, stops, fricatives, affricates, laterals/glides (semivowels) and nasals (as defined in the analysis by Stilp and Lewicki, see Table 1 in Ref [17] for the detailed division of phonemes). We chose not to represent the confidence intervals on *h* because the variations were small (standard deviation of order 0.01).

**Analysis 2**: The purpose of the second analysis is to describe the variations of *β*^⋆^, the optimal value of *β*, on a finer time scale. The motivation behind Analysis 2 is that some phonemes like affricates or stops are subject to acoustic changes even within an occurrence, which can have an impact on the time course of *β*^⋆^ within phonetic units. 400 occurrences were retrieved from the TIMIT database for each phoneme, as in Analysis 1. This time, eight 16 ms slices at regular intervals were considered for each occurrence, possibly with some overlap, instead of a single slice by occurrence. As the occurrences do not have the same duration, the eight steps represent relative time rather than absolute time (1 is the start of occurrence, 8 is the end). The procedure for estimating *β*^⋆^ was the same as described for Analysis 1. This yields 8 values of *β*^⋆^ that represent the temporal evolution of *β*^⋆^. Additional figures on this analysis (e.g. duration histograms) are included in S3 Appendix.

As an additional verification of our methods and results, we also reproduced **Analysis 1** on vowels and nasals using Independent Component Analysis (ICA). The analysis essentially follows the same procedure described in the work by Stilp and Lewicki [17], therefore not detailed here. The main difference of approach is that the regression slopes (*β*^⋆^) were estimated by constraining the regression line to cross the point (*f*_0_, *Q*_0_). This choice was made in order to be consistent with the parametric representation model used in the current study. The description and discussion of this analysis can be found in the appendix S4 Appendix. In the main text, we mention the results of this analysis when they differ from the parametric method, or when they can provide other insights.

### Artificial signals

In parallel to real speech data, we simulated two kinds of synthetic signals to support the interpretation of the results. These signals were analyzed in the same manner as described above. The first kind of synthetic sounds is noises windowed in time or in frequency, with similar structure to consonants. The second kind of synthetic signals simulates sounds emitted by a uniform cylindrical waveguide with different radii. These simulations were conducted so as to illustrate two key factors that are shown to affect the value of *β* in this paper: the localization of noises in time or frequency for consonants (obstruents), and the degree of acoustic radiation at the lips for vowels.

**Simulation 1**: The generated signals were created by applying a Gaussian window on noise in time or frequency. 200 samples of 16 ms were generated with the following procedure. Initially, the noise sounds are samples of Gaussian white noise filtered by a low pass filter of order 1 (cut-off at 2 kHz). Each sample is associated with a parameter *u* ranging from 0 to 1, that controls the time/frequency modulations (*u* = 0: first sample, localized in time, *u* = 1: last sample, localized in frequency). At *u* = 0, the samples of noise are windowed by a Gaussian of time deviation *σ*_*t*_ = 0.01 × *T* = 0.16 ms. At *u* = 1, the noise is convolved by a Gaussian filter (windowed in the frequency space) of frequency deviation *σ*_*f*_ = 0.01 × *f*_*s*_/2 = 80. The sounds of intermediate values make the transition between these two configurations. From 0 to 0.5, they go through time windowing with *σ*_*t*_ increasing. From 0.5 to 1, they are windowed in frequency with *σ*_*f*_ decreasing. *u* = 0.5 is pink noise that goes through little modulation. Some values for the modulation widths are given in Table 1. More details are given in supporting information (S1 File). We also provide an audio file (S2 File) showing the transition between all the samples.

**Table 1:** Correspondence between the control parameter *u* and modulation widths (Simulation 1)/aperture radius (Simulation 2).

*u*	*σ*_*t*_ (ms)	*σ*_*f*_ (Hz)	*r* (cm)
0	0.2	–	0.20 ([Ʊ])
0.2	1.0	–	0.42 ([u], [Ɔ])
0.4	4.5	–	0.64 ([Ʌ], [I])
0.6	–	2000	0.86 ([i], [ɛ])
0.8	–	470	1.08 ([æ])
1	–	80	1.30 ([ɑ])

For reference, we give examples of vowels that have comparable sectional area under the assumption of a circular aperture (see Ref [29] for more precise data).

**Simulation 2**: The second kind of generated signals simulates sounds emitted by a uniform cylindrical waveguide, open at one end, and with different values for the cross-sectional area. The goal is to synthesize vowel-like sounds with various bandwidths. The termination impedance was chosen as for a radiating sphere of radius *r* [27]:

$$\begin{array}{c} Z=\rho_{0}c[\frac{{\left(kr\right)}^{2}}{1+{\left(kr\right)}^{2}}+j\frac{kr}{1+{\left(kr\right)}^{2}}]\;, \end{array}$$ (10)

where *k* = *ω*/*c*, *ρ*_0_ and *c* are resp. air density and the speed of sound. To account for other surface losses in the waveguide, *k* was substituted with *k* = *ω*/*c* − *jα*, $\alpha =1.2e-5\sqrt{\omega }/0.01$ as in Ref [28]. The waveguide length was similar to the length of the vocal tract (in average 16.5 cm). We generated again 200 samples. This time, the parameter *u* ∈ [0, 1] controlled linearly the radius *r* of the cylinder, ranging from *r* = 0.2 (*u* = 0) to *r* = 1.3 (*u* = 1). Some values of *u* and *r* are given in Table 1. See S3 File. for more details on the generation and S4 File. for a sound file showing the transition between all the samples.

For Simulations 1&2, we estimated *β*^⋆^ for the 200 generated samples with a procedure similar to Analyses 1&2. More precisely, after the decompositions and the computation of the scores on each sample were done, we obtained a 200 × 30 score matrix *h*(*u*, *β*). We applied a Gaussian filter (*σ* = 1) on the *u*-axis, then we computed *β*^⋆^ on each row.

## Results

It is recalled that in the following, *β* refers to *β*^⋆^, the exponent of the power law satisfied by *Q*_10_, with respect to center frequency, offering the most sparse decomposition of the data. The results are presented going each time at a finer level of speech, first for non-structured sounds (stops, fricatives, and affricates), then for structured sounds (vowels, semivowels, and nasals). This distinction is justified in the next paragraph. The comprehensive description suggests a level dependence of the fine-grained statistical structure of speech, which is analyzed in the last subsection of Results.

### The distinction between structured and non-structured sounds

**Fig 3:**
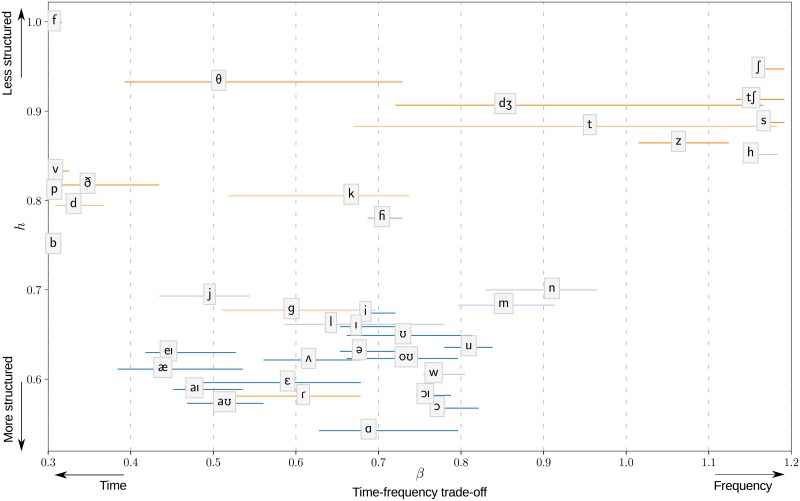
Distribution of American English phonemes in the (*β*, *h*) plane. *β* is the exponent of the power law satisfied by the quality factor with respect to center frequency for the most sparse representation of the data. High values of *h* characterize the lack of structure. The labels are positioned on the means of the bootstrap distribution. The lines represent 70% bootstrap confidence intervals. The bootstrap distributions are based on 400 samples for each phoneme and 3000 repetitions.

The distribution of the values *β* obtained for the broad phonetic categories (Fig 2) is consistent with the distribution of exponents found by Stilp and Lewicki (further compared in Discussion). The addition of the average value of the cost function *h*, reflecting the lack of structure, demonstrates the existence of two separate types of sounds being structured sounds (*h* < 0.7: semivowels, vowels, nasals) and non-structured sounds (*h* > 0.7: stops, affricates, fricatives). The latter are characterized by poor time and/or frequency structure. It does not mean that consonants have no structure at all on a larger time scale (e.g. stops have a clear time pattern closure—burst—opening phase). It means, however, that non-structured sounds are more easily related to noise on small times scale of about 10 ms. The distinction between structured and non-structured sounds is relevant to our analysis because we found that the factors determining *β* are different for each type. They are described separately in the following paragraphs. Most sparse signals are the approximant [ɹ] (*β* = 0.82) and the related vowels [ɚ] (*β* = 0.85) and [ɝ] (*β* = 0.88) with *h* = 0.47 for the three phonemes, not represented in Fig 3. *h* is more than twice larger for the least sparse sounds being the fricatives [f] (*h* = 1.00, maximal value) and [S] (*h* = 0.95).

The phonetic categories are unequal in terms of variability. The bootstrap confidence intervals on *β* reflect the diversity of acoustic features within a category. For example, affricates, which borrow acoustic features from both stops and fricatives, are characterized by large confidence intervals covering almost the entire range of values. The consistency of bootstrap confidence intervals is confirmed in the following paragraphs when the statistical structure of the phonetic categories is described in more details. The figure at the finer level of phonemes (Fig 3) shows that variability can sometimes be explained by opposite values of *β* within a class. Most of the fricatives are in the region *β* > 1, but we find other fricatives ([v], [ð], [f]) in the opposite region *β* < 0.5. Other times, the same variability is observed at the level of phonemes, showing that this variability still exist at finer level. For example, the affricate [dƷ], the fricative [T] or the stops [t] and [k] still have large confidence intervals. Although there is some scattering among the fricatives and stops, phonetic categories form consistent groups. Most of the time, phonemes that are close in the acoustic space are also close in the (*β*, *h*) space. However, the phonetic categories that were used for Fig 2 do not always offer the best clustering of the data for statistical structure. Some phonemes appear to belong to a cluster different from their attributed category. The main examples are the aspirant [h] going with the cluster of fricatives, the fricatives [v] and [ð] with stops, the stop [g] with the laterals [j] and [l], the *flap* [ɾ] with approximants.

Another measure of the significance of *β* is contrast, defined by the relative difference between the maximum and minimum value of *h*. By noting *h*_min_ = min_*β*_ *h*(*β*) and *h*_max_ = max_*β*_ *h*(*β*), contrast *c* is defined by

$$\begin{array}{c} c=\frac{h_{\max}-h_{\min}}{h_{\max}}\;. \end{array}$$

It is small for a flat cost function and large when the cost function has a clear minimum. *c* = 1% for speech as a whole when the scores are averaged over all the samples. The phonetic categories are in increasing order of *c*: affricates (0.4%), stops (0.7%), fricatives (1.7%), vowels and approximants (1.8%), and then nasals (2.1%). Contrast again indicates a strong variability for stops and affricates which requires to be examined at a finer level. This is done in the following subsection.

### Stops, fricatives, and affricates

**Fig 4:**
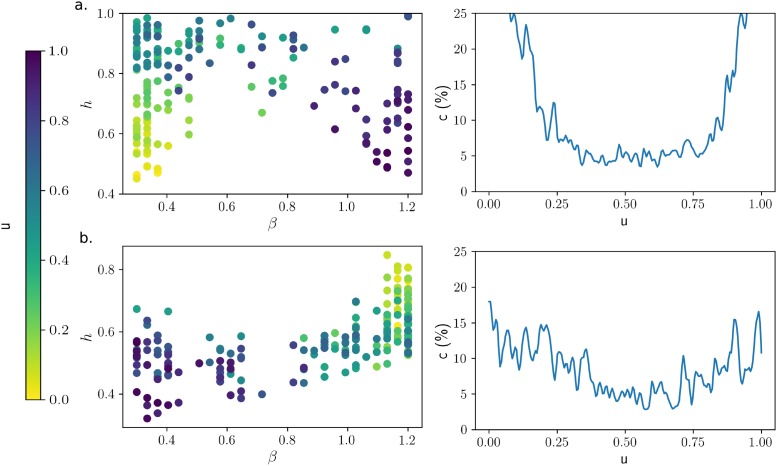
Best decompositions of artifical signals. Scatter plot, left: distribution of generated signals in the (*β*, *h*) plane. Right: contrast against the control parameter *u*. **a. Simulation 1**: Modulated noises from time windowing (*u* = 0) to frequency windowing (*u* = 1). Samples at *u* = 0.5 are neither modulated in time nor in frequency (white noise). This simulation shows that *β* is related to localization for noise sounds: time localization corresponds to a minimal *β* value (as for stop onsets), frequency localization corresponds to a maximal *β* value (as for sibiliant fricatives). **b. Simulation 2**: Radiated sounds at the output of a uniform cylindrical waveguide with different radii, from *r* = 0.2 *cm* (*u* = 0) to *r* = 1.3 *cm* (*u* = 1). Greater apertures means greater losses, wider bandwidths, hence lower values of *β*.

Stops, fricatives, and affricates are non-structured sounds (associated with the highest values of the cost function *h*), meaning that they are more easily assimilated as noise. Gaussian noises windowed in time or in frequency (Simulation 1) provide a coarse model for non-structured sounds. Gaussian white noise maximizes entropy for fixed output power. In the case of non-structured sounds, the coding strategy amounts to reduce the noise in the output components, by decreasing the mean amplitude value. In the ideal case of a decomposition that maintains total power throughout the transformation, the most efficient strategy is to have filters with an almost zero output and filters with maximum response. This strategy increases the sparsity of the signal decomposition as it puts as many outputs as possible with zero response. More generally, the optimal decomposition of noise sounds shifts toward a time (or frequency) decomposition if it has a sharp power increase/decrease in the time (or frequency) domain. We illustrate this fact with Simulation 1 on modulated noises (Fig 4A). *β* takes the lowest value (time representation) when the noise is multiplied by a Gaussian function localized in time. Then, *β* increases up to a median value as the Gaussian expands. At the same time, *h* increases because any structure is lost. Halfway through the simulation, at *u* = 0.5, the generated samples lose most structure, and *h* is maximum. *β* has a rather erratic behavior and the score function becomes flatter as indicated by the low contrast value. The symmetrical pattern takes place when the simulation goes on frequency modulations. At *u* = 1, *β* takes the highest value (frequency representation). For a stop, the modulation function can be thought as a gate function in the time domain with random time for the burst. This is close to simulation with u = 0 when the modulated noises show a rapid increase in intensity for a short amount of time. Based on this very simplified model, we expect the time representation to be optimal. Stops are indeed associated with low *β* values (Fig 3 from Analysis 1), but the large bootstrap confidence intervals indicate a more complex behavior described further on.

Fricatives are more explicit for now with Analysis 1 because they can be well approximated by stationary processes. Fricatives are the result of turbulent airflow occurring at a constriction in the vocal tract. The noise produced at the place of constriction is then filtered by the vocal tract, similar to the generated sounds passing through frequency modulations in Simulation 1 (*u* > 0.5). Most fricatives are characterized by values of *β* close to 1 consistent with this frequency description (Fig 3). It is at least true for the sibilant fricatives (*β* > 1). Sibilant fricatives are filtered with a short cavity after the alveolar ridge and therefore present sharp increase/decrease of power in the high frequency range. It is a clear trend for the hissing alveolar fricatives [s] (*c* = 4%) and [z] (*c* = 3%) and it remains valid for the hushing post-alveolar fricative [S] (*c* = 1%). However, the wide-band labial or dental fricatives [f], [T], which are less affected by vocal filtering, are associated with lower *β* values (*c* = 1%, *c* = 0.6%, resp.) and maximal *h* values (poor structure). Voiced fricatives are an intermediary case for what has been seen as they are affected by both time and frequency modulations. In addition to vocal filtering, the sound intensity follows the repeated openings and closures of the glottis. The coincidence of time and frequency modulations is likely to explain the shift to bottom-left on the (*β*, *h*) plane when replacing the unvoiced versions of the fricatives by the voiced ones (compare [s], [h], [T], [f] with [z], [ɦ], [ð], [v], resp.).

**Fig 5:**
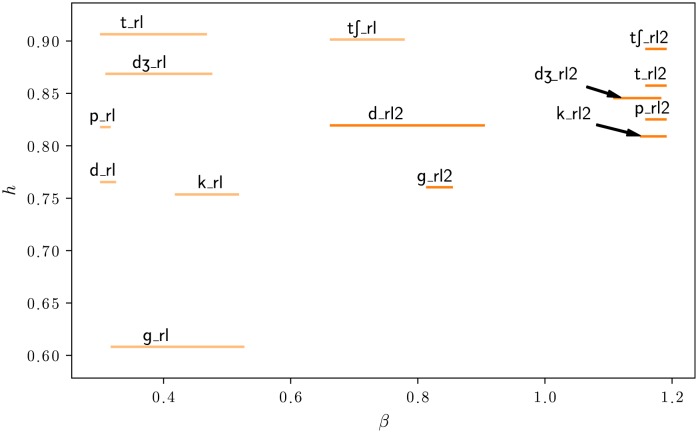
Detailed distribution of stops and affricates in the (*β*, *h*) plane. When stop or affricate releases are separated into two parts of same duration, first parts _*rl* (including the bursts) are best represented in a dictionary with a low *β* value (time representation) but second parts _*rl*2 are best represented in a dictionary with a high *β* value (frequency representation), similar to fricatives. The figure shows the means of the bootstrap distributions and the 70% bootstrap confidence intervals.

The description of stops and affricates must be refined to take into account dynamic aspects. Stops and affricates change in time following the pattern closure—release burst—release transition. During the closure, no sound is emitted (or a low frequency sound only as in [b]). Closures were always ignored in the analyses because they do not contain high frequency information. After the closure, the release can be divided into two phases. The first phase is the burst following the instant of the occlusion release. During this phase of small duration (few milliseconds), the intensity increases and decreases rapidly and the power spectrum is typically flat. In the second phase—the opening or aspiration phase—there is still some obstruction at the place of articulation and/or aspiration at the glottis, resulting in sounds similar to fricatives [30]. We conducted a specific analysis to determine if the biphasic nature of stops and affricates has an impact on the parameter *β*. We performed the procedure described Analysis 1, but this time we separated the releases into two parts of equal duration. The results, reported in Fig 5, show that the dual nature of stops and affricates is also revealed by *β*. While the first parts containing the bursts (suffix _*rl*) are characterized by minimal values of *β*, the second parts (suffix _*rl*2) are characterized by higher values close to 1. Values for stops as a category are also reported in Fig 2 with the same suffixes. This analysis shows that the opening phase of stops is in reality similar to fricatives with regard to statistical structure.

**Fig 6:**
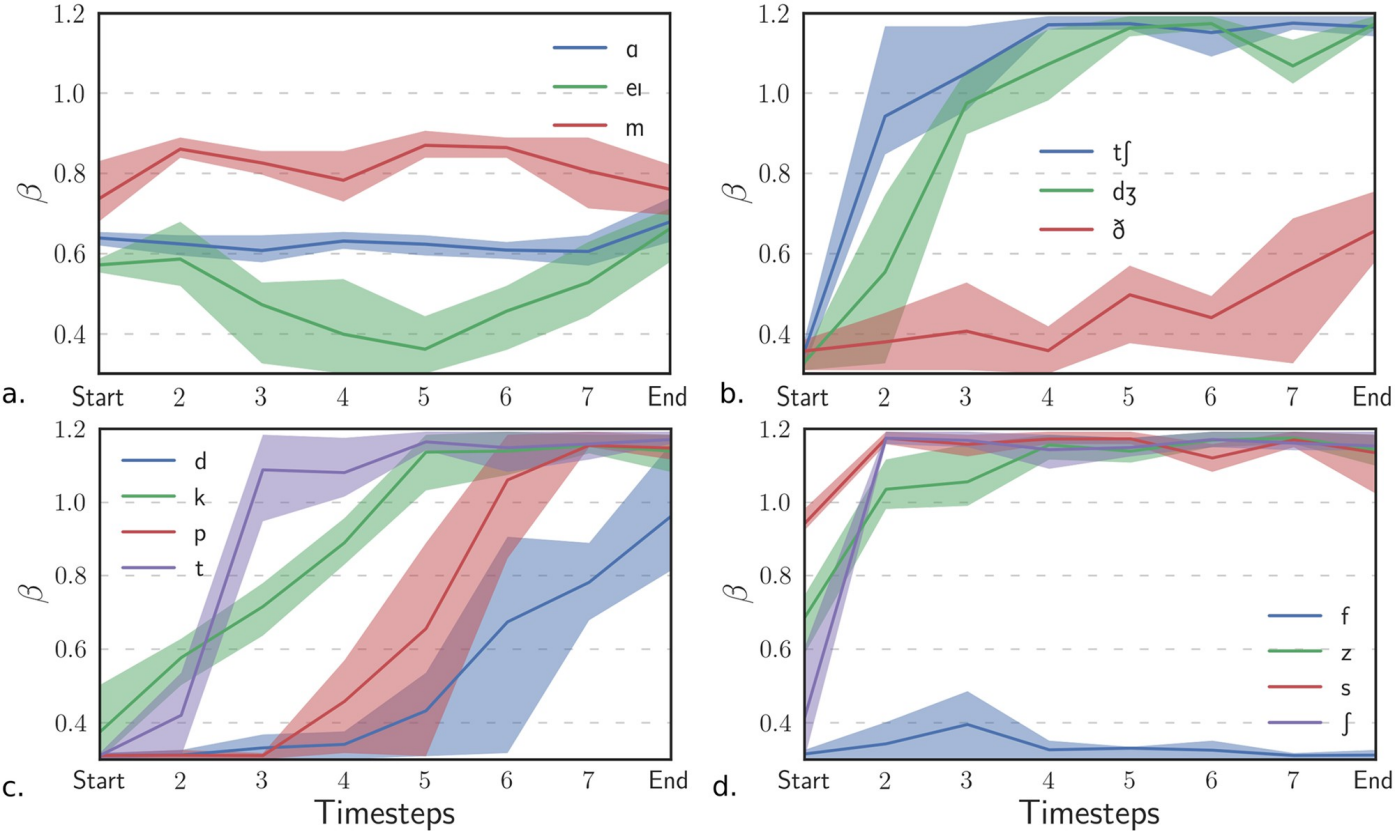
Temporal evolution of *β* for some phonemes. The time steps represent the relative times between the beginning (*Start*) and end (*End*) of the occurrences at regular intervals. The region filled represents 70% confidence intervals. Stop onsets are associated with minimal values of *β* (time representation), but the opening phases are associated with higher values. **a**. Vowel: A, diphtong: eI, nasal: m **b**. Affricates: tʃ, dʒ, fricative: ð **c**. Stops: d, k, p, t **d**. Fricatives: f, z, s, ʃ.

We can describe further the temporal behavior of *β* for stops and affricates with the results of Analysis 2. Fig 6 provides the time evolution of *β* within some phonemes. The parameter *β* is stable for vowels or nasals—apart from diphthongs—but it increases during the occurrences of stops and affricates, joining the extreme values. This behavior is consistent with the description in the previous paragraph. However, this transition is more or less abrupt depending on the nature of the opening phase. The stop [t], whose opening phase is similar to the sibilant fricative [s], has a fast transition after the burst. In contrast, the stop [p] has a more gradual transition. We explain this gradual transition by the fact that the opening phase of the stop [p] is similar to the low *β* fricative [f] on which some formant structure appears gradually when the back cavity plays a role again (as for the high *β* fricative [h]). Although the change is less pronounced, fricatives have also an upward shift of *β* at their onset.

### Vowels, semivowels, and nasals

The reasoning in the previous section cannot apply apply to structured sounds, in particular vowels. For these sounds, structure has to be made explicit.

The structure of vowels can be seen both in time and in frequency. Along the frequency axis, vowels are characterized by spectral peaks arranged at intervals of about 1 kHz, corresponding to the resonances of the vocal tract (formants). However, we make the remark here that the harmonics of *F0* are not resolved on the time scale we consider, and therefore do not play any role on the statistical structure in our analysis. On the time axis, the signal presents peaks of intensity at the instant of glottal closure remaining true if the signal is band-passed around formants. The latter statement stands at least for the first formants as higher formants can be excited at other instants, especially at the glottal opening [31]. On one glottal cycle, a naive image of the underlying structure in the time-frequency plane can be a comb shape whose “teeth” represent the formants. The complete structure cannot be perfectly covered by a single Gabor filter bank: a representation is always a compromise between time and frequency favoring either the glottal pulse or the tails of the formant oscillations. From a frequency point of view, it means that either the wideband parts of the signal associated with low response and low group delay, or the narrow bands associated with the formants, are favored. This competition between the pulse and the formant oscillations has a visible effect on the quality factor of the efficient coding filters at medium frequencies, especially between 1 and 2 kHz. When ICA is performed on back vowels, which have low second formants, the quality factor increases at 1 kHz more steeply than for front vowels (see Fig 1 of Ref [17]). This effect is also visible when ICA is conducted on single phonemes (as reported in S4 Appendix).

The optimal representation, regardless of the point raised in the previous paragraph, is expected to be related to formant bandwidths. Formant bandwidths are determined by the level of damping, hence by acoustic losses, in particular wall losses at low frequencies and radiation losses at high frequencies [32, 33]. Since higher formants play a greater role in the determination of *β*, a key factor for the statistical structure of vowels is the degree of acoustic radiation at the lips. The level of acoustic radiation depends on the termination impedance, which increases with frequency and lip opening [27, 34]. Simulation 2 shows the impact of aperture radius on *β* with synthetic vowels generated by simulating a uniform cylindrical waveguide (Fig 4B). We obtain a maximal value of *β* with the smallest aperture (*u* = 0), associated with low damping and narrow bandwidths, but we get the opposite for a larger aperture (*u* = 1). The two end points are separated by a steep phase transition occurring between u = 0.5 and u = 0.7 (r = 0.70.9). The correction of the wave number that we used in the simulation corresponds to a low estimate of surface losses. Hanna and al. proposed instead to increase this correction by a factor 5 [28]: we found that this change makes the transition to be close to *u* = 0. The estimation of acoustic radiation with aperture radius is an approximation, in particular it does not take into account inner reflexions [32]. The same trend can still be observed for real data although the transition is less pronounced. The vowels form a tight cluster around *β* = 0.6 ± 0.2, but the unrounded vowels and diphthongs [æ], [ε], [eI], [aƱ], and [aI] yield lower *β* values than the rounded vowels [u] and [Ʊ] (Fig 3). The intermediate sounds [ɔ], [ə], [ʌ], [i] and [I] are found in between. The vowel [α], however, does not match the rest of the distribution—this may be the consequence of the constriction at the back of the vocal tract weakening the effect of acoustic radiation at the lips. This analysis on vowels has been reproduced using Independent Component Analysis (ICA). The results, shown in S4 Appendix, confirm the trend described above. The largest differences in the *β* values are found for the low back vowels [ɔ] (ICA: 0,68, parametric method: 0.78) and [α] (ICA: 0.63, param.: 0.7). ICA also demonstrates that the biggest disparity in quality factors between vowels is found in the region 1–5 kHz. The analysis on generated samples revealed the other trend that *h* decreases at the same time as *β* (Fig 4B). The most likely reason for this phenomenon is that narrow bandwidths (high *β* values) fill the time-frequency domain with longer tails while damped signals (low *β* values) are localized in time, therefore sparser. However, the trend is not sufficiently clear on phonemes to conclude that this rule applies to real data.

The nasals are found in the continuity of the vowels, with higher values of *β* and *h*, meaning that nasals are better adapted to a frequency decomposition. We explain this fact by the presence of antiresonances surrounding the formants which have the effect of cutting the bandwidths of the nasals. An element that supports this argument is that we find high quality factors close to *f*_*c*_ = 3 kHz when ICA is applied to the [m] sound (S4 Appendix). This frequency is associated with the second antiresonance of the mouth cavity. The above explanation is rather contrary to the known fact that nasals have wider bandwidths due to greater surfaces losses. This is not contradictory since the region of interest is in the high frequency range and the values of wide bandwidths (e.g. 10dB bandwidths) are more significant here than the usual narrower 3dB bandwidths. The estimates of *β* using ICA, although in the upper part of the cluster of vowels and nasals, are smaller than those obtained by the parametric method, particularly for the [n] sound (ICA: 0.75, param.: 0.92).

Semivowels are within the same range of values of *β* and *h* as vowels. The rhotic approximant and r-colored vowels occupy the lower right part of the cluster (*β* = 0.8, *h* = 0.47) in the (*β*, *h*) plane. A possible explanation for the low score *h* for r-sounds is that they present a strong frequency decrease in high frequencies, hence the underlying structure for the high-passed filtered signal is essentially a prominent peak in frequency close to 1 kHz.

### Level dependence of the statistical structure of speech

**Fig 7:**
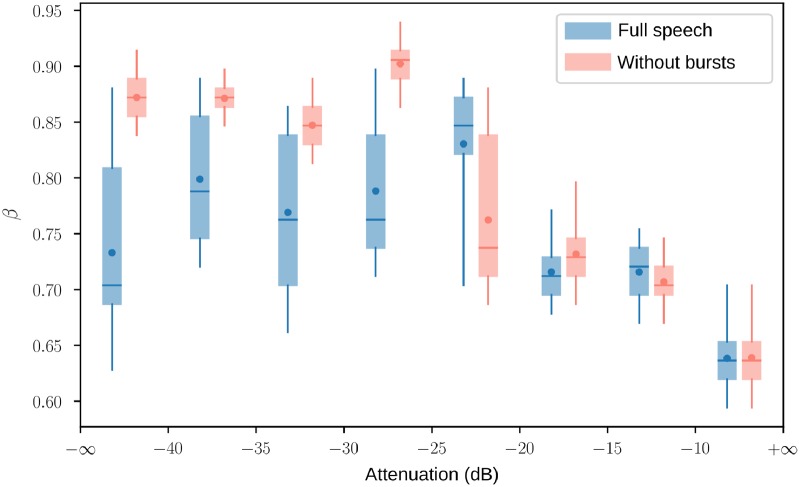
Exponent *β* with respect to intensity level. Exponent *β* associated with the most sparse decomposition of speech sounds of same intensity, in 5dB intervals (ref:max). **In blue**: Full speech, **in red**: same but with the first parts of stop and affricate releases removed. The box plots show the quartiles, the [5%, 95%] percentiles (whiskers) and the mean (dot) of bootstrap distributions obtained from 2 500 16 ms-slices of speech. Contrast is under 1% before the knee and above after.

The above-described variations in statistical structure are effectively summarized by describing the level dependence of the parameter *β*. This analysis is also significant because the frequency selectivity of the inner ear changes with sound intensity level (see in Discussion: Agreement with the efficient coding hypothesis and cochlear signal processing). Fig 7 shows the value of *β* as a function of intensity level by intervals of 5dB. The left part of the figure corresponds to the lower sounds of speech, which are mainly the non-structured sounds (Stops, fricatives, and affricates). We have seen that, at least if we ignore the onsets of plosives and affricates, non-structured sounds are better decomposed with a value of *β* close to 1, congruent with the plateau observed in Fig 7 when these parts are removed. The right part of Fig 7, corresponding mostly to vowels, is characterized by a decrease in *β*. This can be explained by the fact that, for vowels, formant bandwidths and sound intensity increase simultaneously with the opening at the lips. In support of this explanation, we reproduced Fig 7 with the synthetic vowels generated by Simulation 2. We found the same decrease in *β* with sound intensity level, however with a steeper transition (S1 Fig).

## Discussion

### Distribution of the parameter *β* for phonetic categories

The overall distribution of *β* values for the broad phonetic categories (Fig 2) is in agreement with the regression slopes of *Q*_10_ on *f*_*c*_ (on log-log scale) found by Stilp and Lewicki using Independent Component Analysis. In particular, the slope *β* is found between 0.7 and 0.8 for speech data as a whole, with both ICA and our method. The most noticeable gap is for nasals (0.9 compared to 1.05 in Stilp and Lewicki [17]). However, when the regression line is constrained to cross the point (*Q*_0_ = 2, *f*_0_ = 1 kHz), in accordance with our setting, ICA generates *β* estimates that are lower than those found by Stilp and Lewicki for nasals and vowels, and consistent with the parametric estimation (the correlation coefficient is r = 0.90 between the *β* values found using each method on vowels and nasals). The discussion of these results is developed in S4 Appendix. The exact value of *β* with the parametric method depends on several experimental settings, in particular: the values of *f*_0_ and *Q*_0_, the preprocessing of data, the weighting strategy. However, we have found that the distribution of *β* values, and its interpretations in terms of statistical structure, are robust to changes in these parameters.

Phonemes that are close acoustically were found together in the (*β*, *h*) plane, showing the consistency of *β* with acoustic properties. The phonetic categories that were used by Stilp and Lewicki (reused for Fig 3) can still be adjusted marginally. For example, the aspirant [h] belongs to the cluster of fricatives rather to the cluster of semivowels.

### Relationship between the parameter *β* and acoustic features

In the Results section, we inferred the main acoustic factors that affect the *β* parameter based on the distribution of *β* values at the level of phonemes (or below). Some of these properties coincide with previous proposals, but others are new or clarify some previous ideas.

In 2002, Lewicki examined whether the spectral tilt—the natural decrease of power spectrum density—could explain the power law satisfied by the quality factor [11]. His conclusion was that there is no connection between the two. The average power spectrum density indeed has a low impact on signal structure, because the efficient coding filters are localized in frequency—it has an effect on the weighting between midrange and high frequencies but not on the atomic components. An exception is that the addition of a decrease or increase in the power spectrum leads to the emergence of frequency structure in the case of non-structured sounds. This reasoning applies in particular to fricatives: the high-pass filtered hissing sounds [s] and [z] are associated with a higher value of *β* compared to fricatives with broadband spectrograms (e.g. [f]).

In 2013, Stilp and Lewicki listed three others acoustic factors that could affect the value of *β*: *harmonicity*, *acoustic transience* and *bandwidths* [17]. We argue that the *F0* periodicity plays little if no role because the efficient coding filters are shorter than the period length. Therefore, *harmonicity* in the usual sense (*F0* harmonics) does not add any frequency structure on this time scale (unlike formant structure). More generally, acoustic changes of characteristic time greater than the duration of a glottal cycle (e.g. coarticulation, formant transitions) do not have a significant impact on the efficient coding filters as such. We have found that an acoustic factor more significant than harmonicity for the statistical structure of speech is *voicing*. The fact that voiced sounds are characterized by scarce time-localized excitations has the effect of enhancing time localization, and decreasing both *β* and *h*. Consequently, vowels have been shown to be associated with relatively low values of *β*, a result that could appear counterintuitive. Vowels are sustained sounds that are often believed to be better captured by a frequency representation. This view might be biased by the source-filter model that focuses on the resonances in the frequency space and makes extensive use of Fourier analysis. The analysis of the statistical structure of speech supports the opposite view that a time decomposition, i.e. characterized by a low quality factor, would be more appropriate for the efficient coding of vowels. This work reinforces the view that *transiency* is a key acoustic factor for the statistical structure of speech, since the lowest values of *β* are reached for stop bursts because of the sudden increase in intensity. It also supports the hypothesis that *β* is related to *formant bandwidths* for vowels and nasals. It suggests that, for these phonemes, two key acoustic factors are the degree of acoustic radiation at the lips and the existence of antiresonances. A factor related to the degree of acoustic radiation is vowel openess (but more specifically lip opening). The value of *β* is decreased by greater opening but increased by antiresonances, however these two parameters alone do not explain the entire distribution of values for vowels and nasals.

Because *β* is tied to acoustic factors, the description of the statistical structure of speech could be extended to non-speech sounds (animal vocalizations and other environmental sounds). However, this assertion will have to be verified by a separate analysis.

### Agreement with the efficient coding hypothesis and cochlear signal processing

Early in the study of the efficient coding of speech using ICA, a parallel was drawn between the theoretical optimal decomposition of speech and cochlear tuning [11]. Estimates of the frequency selectivity of the inner ear based on physiological measurements in mammals are consistent with the power law model used in this work [35, 36]. The exponent is about the same than for ICA filters, although slightly lower (0.6 compared to 0.7-0.8). This agreement is a replication in the field of audition of a result in visual neuroscience: ICA or algorithms of sparse coding applied to natural images produce oriented Gabor wavelet-like filters similar to the receptive profiles in the primary visual cortex [8, 37]. Speech, however, is special in that it is a human-controlled stimulus, even if it is subject to acoustic constraints. At the time of the first analyses of speech using ICA, Lewicki proposed the hypothesis that speech evolved to be optimally coded by the auditory system, rather than the contrary. The specificity of human auditory tuning is still a subject of controversy. While it has been argued that it is not very different from unspecialized mammals [38], there is increasing evidence that frequency selectivity is significantly higher in humans [36, 39], especially in response to low intensity sounds [40]. Lewicki also suggested that an explanation for the median *β* value is the right balance between transient and sustained sounds in speech. The same agreement with physiological data was obtained with a mixture of environmental sounds and animal vocalizations [11]. The scattering of *β* when ICA is performed on subclasses of speech could however imply a more efficient coding scheme. Stilp and Lewicki suggested that the distribution of values is congruent with the diversity of time-frequency trade-offs of the characteristic responses found in the cochlear nucleus [17]. But they admitted that these observations for the neurons of the cochlear nucleus stand for single tones but maybe not for complex stimuli. It can be added that the recombination of filters subsequent to the cochlear decomposition is a compute intensive task, difficult to integrate in an efficient coding scheme.

Instead, we argue that if an efficient coding strategy is implemented in the auditory system to adapt the neural representation to the input, it should be achieved at the periphery. The assumption that the auditory filters are fixed does not reflect the actual behavior of the inner ear. Due to compressive nonlinearities in the cochlea, the shape of the auditory filters changes with the input signal. As a first approximation, this nonlinearity can be characterized by a level dependence, with a broadening of filter bandwidths when sound intensity level is increased. This nonlinearity is stronger in high frequencies [41–43], consistent with the initial assumption that the variations in the quality factor are small at 1 kHz but large at 8 kHz. The description of the fine-grained statistical structure of speech shows that an agreement with nonlinear cochlear signal processing is plausible. The *β* parameter is indeed negatively correlated to sound intensity (Fig 7), at least if the first parts of stops and affricates containing the bursts are ignored. However, it would require further investigation to determine the extent to which this agreement is valid, and whether the specificity of onsets reflects one aspect of the temporal processing of the inner ear (e.g. short-term adaptation).

One of the limitations of comparisons with the auditory system based on Gabor filters is that cochlear filters are not symmetric in the time domain [44]. The filters at the output of ICA do not present a strong asymmetry, but asymmetric filters can be obtained if sparse response patterns are reinforced by a matching pursuit algorithm [12]. Another limitation of the method used in this study is that the results of ICA can depart from the power law model when considering specific classes of speech sounds (some cases have been described in S4 Appendix). The parametric model is still convenient because speech sounds can be compared at a fine level with a single parameter. Including the intensity level as a control parameter provides a simple strategy to adapt dynamically the coding to the input, in addition reflecting the nonlinear behavior of the cochlea. An adaptive code based on phonemes would not be feasible in practice because it would take too long to recognize a phoneme before the representation could be adjusted, on the contrary the sound intensity level is a parameter that can be captured instantaneously. However, the fine-grained statistical structure of speech, as described in this article, is only one aspect of the regularities present in the speech signal. In particular, the correlations involved in the determination of *β* are under 10 ms, but speech coding systems have also to exploit regularities on higher time scales to be fully efficient.

## Conclusion

This work showed that a parametric approach, based on dictionaries of Gabor filters and a sparsity score, can be used instead of ICA to investigate the power laws characterizing the frequency selectivity of efficient speech decompositions. The power-law exponent, the *β* parameter, provides a rich interpretation of the fine-grained statistical structure of speech. The analyses, based on real and simulated data, made explicit the relationships between the exponent and the acoustic features of speech. The key acoustic factors were enumerated according to the dichotomy between structured and non-structured sounds. For non-structured sounds (obstruents), which can be related to noise, the value of the exponent is explained by the localization of intensity or spectral power. Among non-structured sounds, stops and affricates have been shown to be biphasic after the closure: the transient part (burst) is better captured by a time representation, but the end of the release is a fricative-like sound better captured by a frequency representation. For structured sounds, mainly vowels, the power laws are related to formant bandwidths and the degree of acoustic radiation, partly determined by lip opening. The analysis predicted that the exponent should be negatively correlated with sound intensity to be adapted to speech statistics. Cochlear frequency selectivity in mammals also follows a power law whose exponent decreases with sound intensity level; hence, the present study suggests a connection between nonlinear cochlear filtering and the fine-grained statistical structure of speech. Further analyses will have to be carried out to determine whether the efficient coding hypothesis can be extended for peripheral auditory coding.

## Supporting information

S1 Appendix Distribution of American English phonemes in the (β, h) plane for the different weighting strategies. (PDF)

S2 Appendix Bootstrap distributions on the parameter *β* for various phonemes. (PDF)

S3 Appendix Temporal evolution of *β* within phonemes. Temporal evolution of *β* for various phonemes and additional information (duration of occurrences, intensity, *h* value, contrast).
(PDF)

S4 Appendix Analyses of vowels and nasals using Independent Component Analysis (ICA). Complementary analysis and discussion for the estimation of *β* for single phonemes (vowels and nasals), based on ICA.
(PDF)

S1 Data Scores of speech samples. Minimal data for the data analyses. It contains the decomposition scores of 30k speech samples.
(ZIP)

S1 Fig *β* as a function of intensity level for artificial vowels. (PDF)

S1 File Details on the generation of modulated noises. A IPython notebook for the generation of modulated noises (Simulation 1).
(IPYNB)

S2 File Audio file showing the transition between the sounds generated with Simulation 1. (WAV)

S3 File Details on the generation of vowel-like artificial sounds. A IPython notebook for the generation of vowel-like artificial sounds (Simulation 2).
(IPYNB)

S4 File Audio file showing the transition between the sounds generated with Simulation 2. (WAV)
