# Supplementary material for: Fine-grained statistical structure of speech
Source: PLoS One. 2020 Mar 20;15(3):e0230233. doi: 10.1371/journal.pone.0230233 (PMC7083313; doi:10.1371/journal.pone.0230233)
Supplement: S1 Appendix — (PDF) [file pone.0230233.s001.pdf]

Distribution of American English phonemes in the (β, h) plane for the different weighting strategies

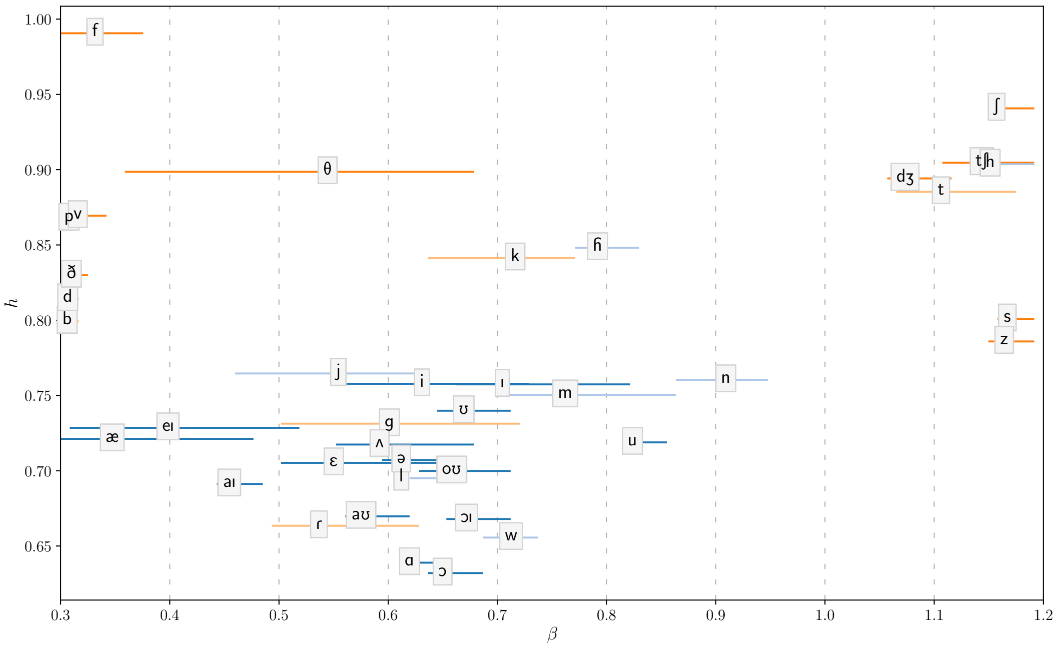

Figure 1: Distribution of American English phonemes in the (β, h) plane with *Strategy A*: raw scores (no spectral whitening). Labels are positioned on bootstrap distribution averages, lines represent 70% bootstrap confidence intervals. Bootstrap distributions are based on 400 occurrences for each phoneme and 3000 repetitions. Not represented: r, ɝ, ɚ ($\beta = 0.93, h = 0.58$).

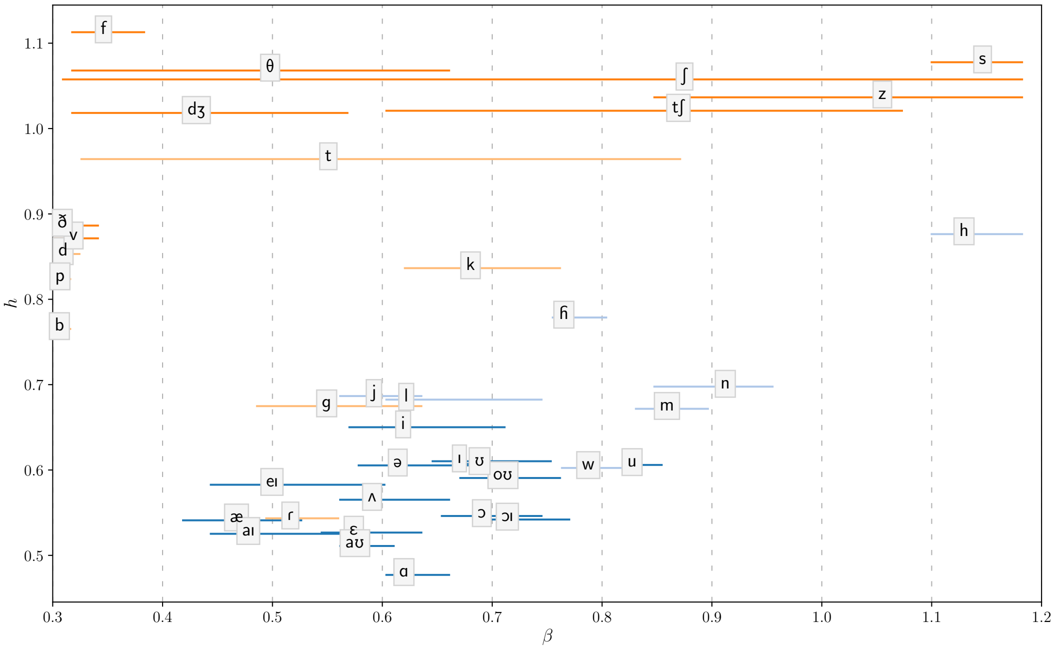

Figure 2: Distribution of American English phonemes in the (β, h) plane with *Strategy B*: spectral whitening +5dB/octave on weights. Labels are positioned on bootstrap distribution averages, lines represent 70% bootstrap confidence intervals. Bootstrap distributions are based on 400 occurrences for each phoneme and 3000 repetitions.
Not represented: r ($\beta = 0.86, h = 0.37$), ɝ($\beta = 0.91, h = 0.36$), ɚ ($\beta = 0.84, h = 0.37$).

Figure with *Strategy C* (slighter gain +2.5dB/octave): in article.
