# Supplementary material for: Fine-grained statistical structure of speech
Source: PLoS One. 2020 Mar 20;15(3):e0230233. doi: 10.1371/journal.pone.0230233 (PMC7083313; doi:10.1371/journal.pone.0230233)
Supplement: S2 Appendix — (PDF) [file pone.0230233.s002.pdf]

Bootstrap distributions

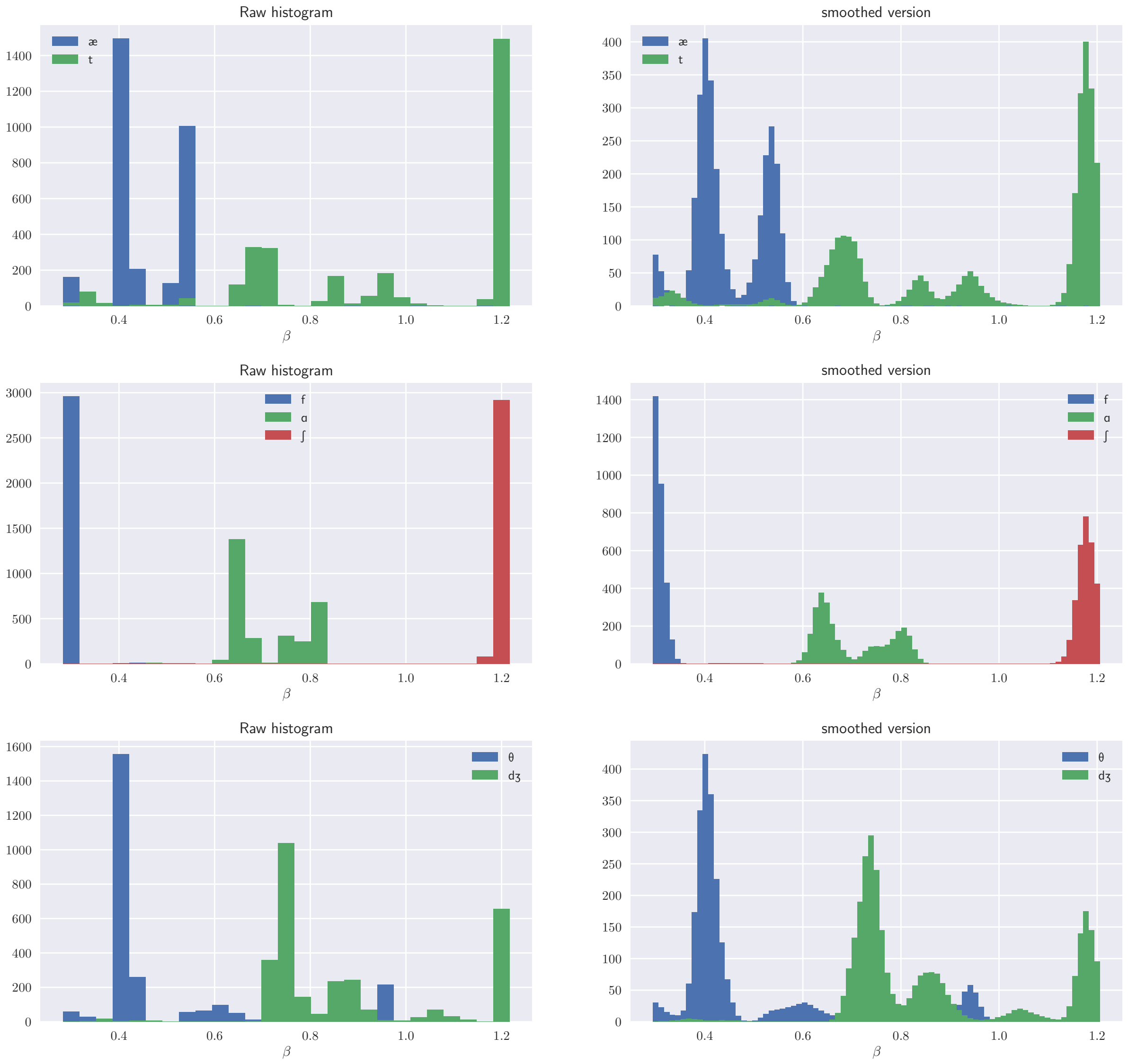

Figure: Bootstrap distributions for phonemes æ,t (up), f, ɑ, ʃ (middle), θ, and dʒ (bottom). The procedure is the so-called smoothed percentile bootstrap. $\beta^{\star}$ is computed after resampling with repetition of 400 slices from the TIMIT database (this is done 3000 times). This results in a first histogram (left) that is smoothed with a Gaussian filter ($\sigma = 0.015$) to obtain the final bootstrap distribution (right). The mean value and the bootstrap confidence intervals are then extracted from the distribution (ex: to get the 70% CI, 15% of the samples on the left and right are excluded).
