# Supplementary material for: Fine-grained statistical structure of speech
Source: PLoS One. 2020 Mar 20;15(3):e0230233. doi: 10.1371/journal.pone.0230233 (PMC7083313; doi:10.1371/journal.pone.0230233)
Supplement: S3 Appendix — Temporal evolution of β for various phonemes and additional information (duration of occurrences, intensity, h value, contrast). (PDF) [file pone.0230233.s003.pdf]

Temporal evolutions of β

Top left: Duration histograms for the occurrences retrieved from the TIMIT database (absolute time), **top center:** intensity I (dB) vs time, **bottom left:** entropy score h vs time, **bottom center:** contrast c vs time, **right:** exponent β vs time.

Vowels and Diphtongs

ɑ

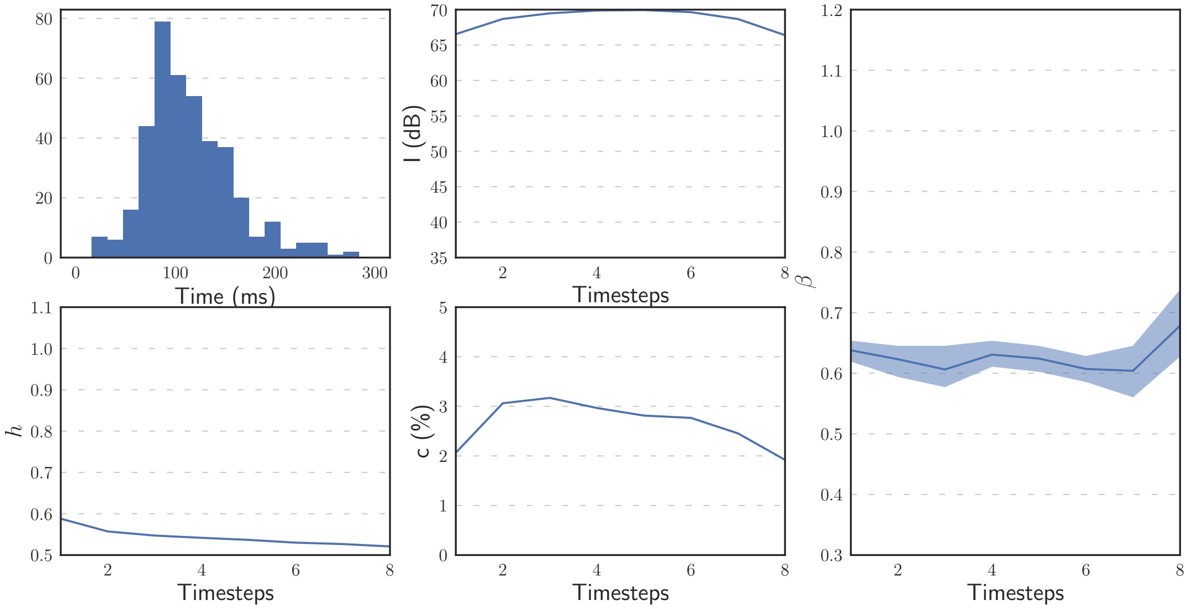

mean duration : 116.1 ms (std : 49.9 ms)

aɪ

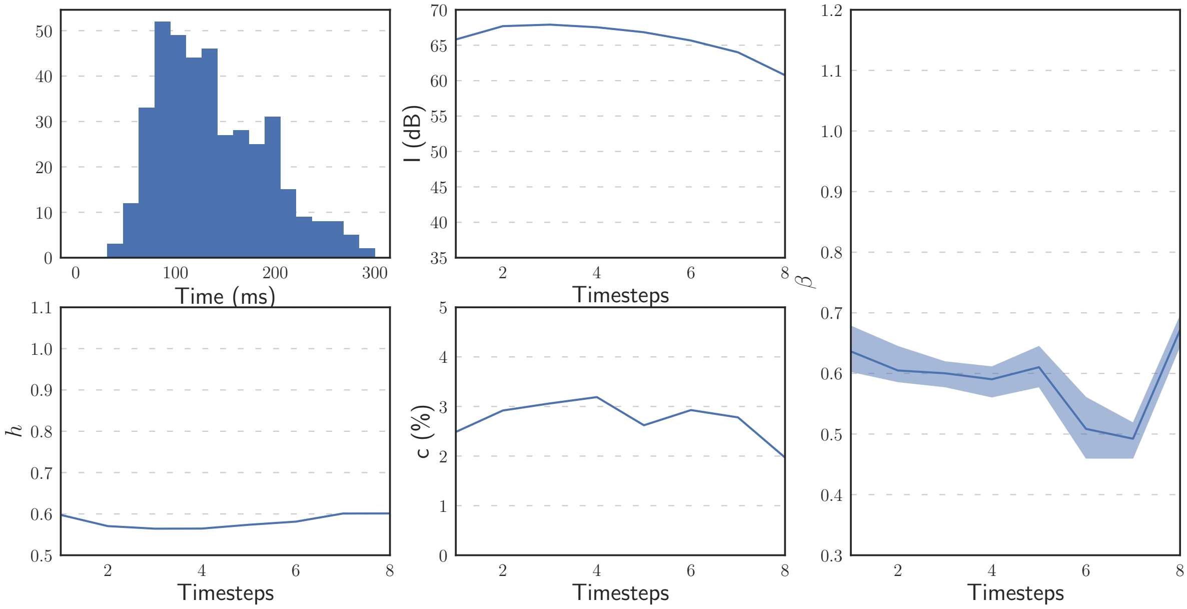

mean duration : 139.1 ms (std : 56.6 ms)

eɪ

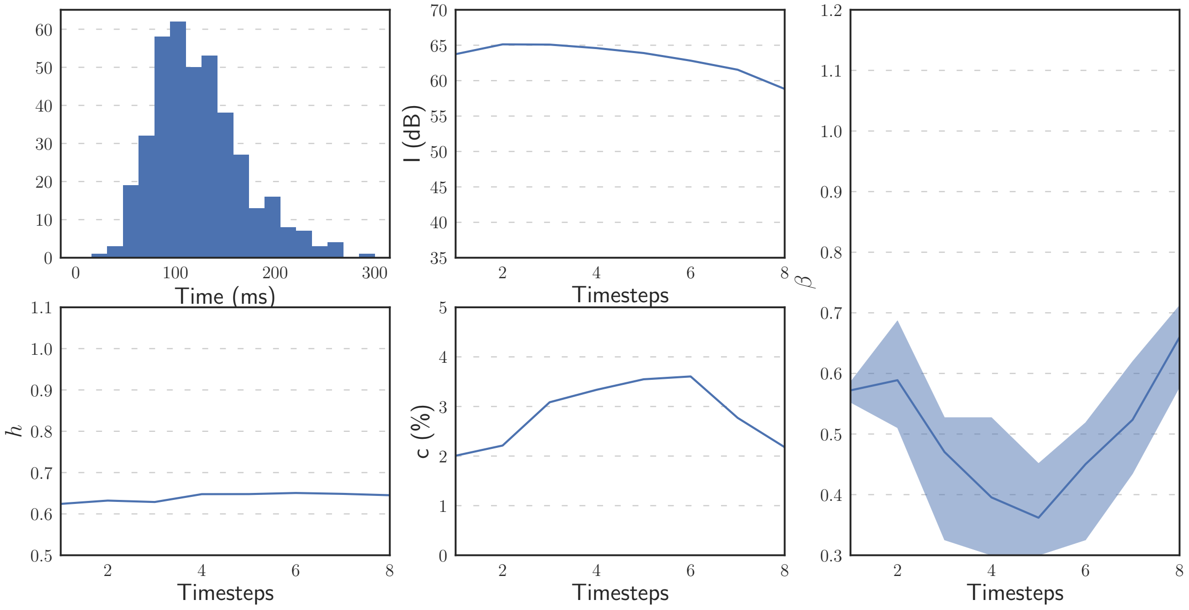

mean duration : 126.7 ms (std : 50.8 ms)

Nasals

m

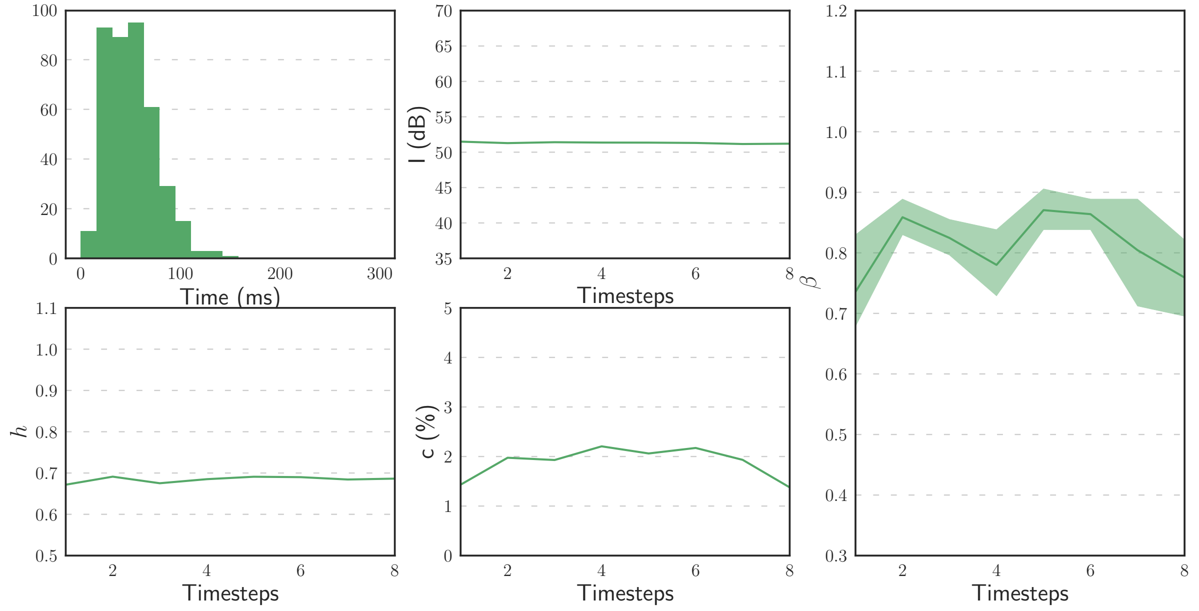

mean duration : 50.9 ms (std : 24.6 ms)

n

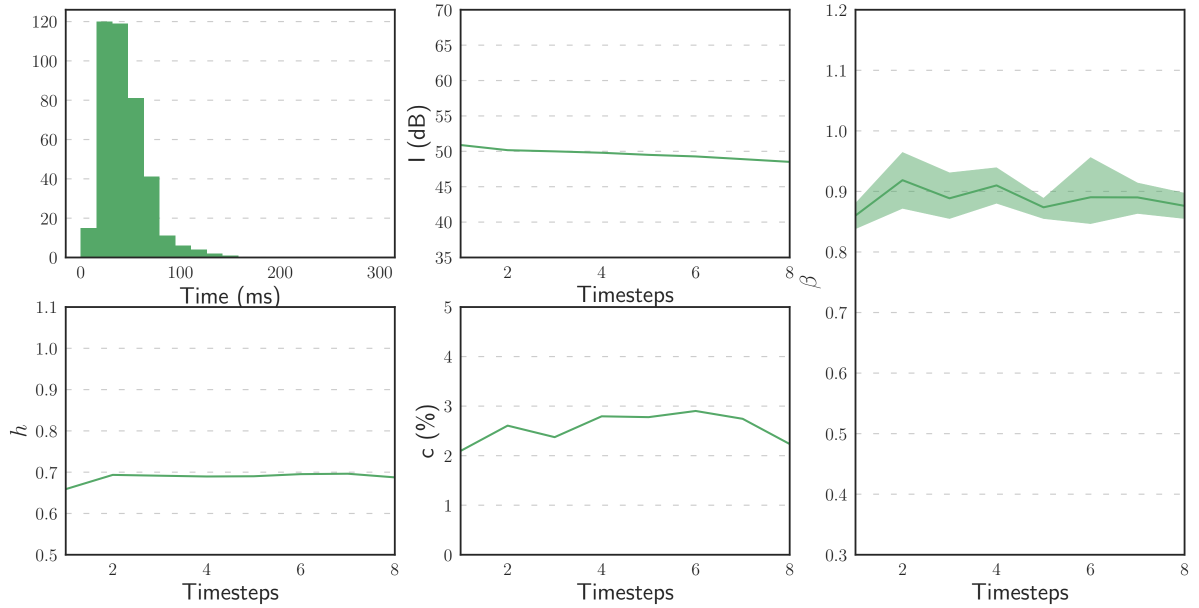

mean duration : 44.0 ms (std : 22.2 ms)

Stops

d

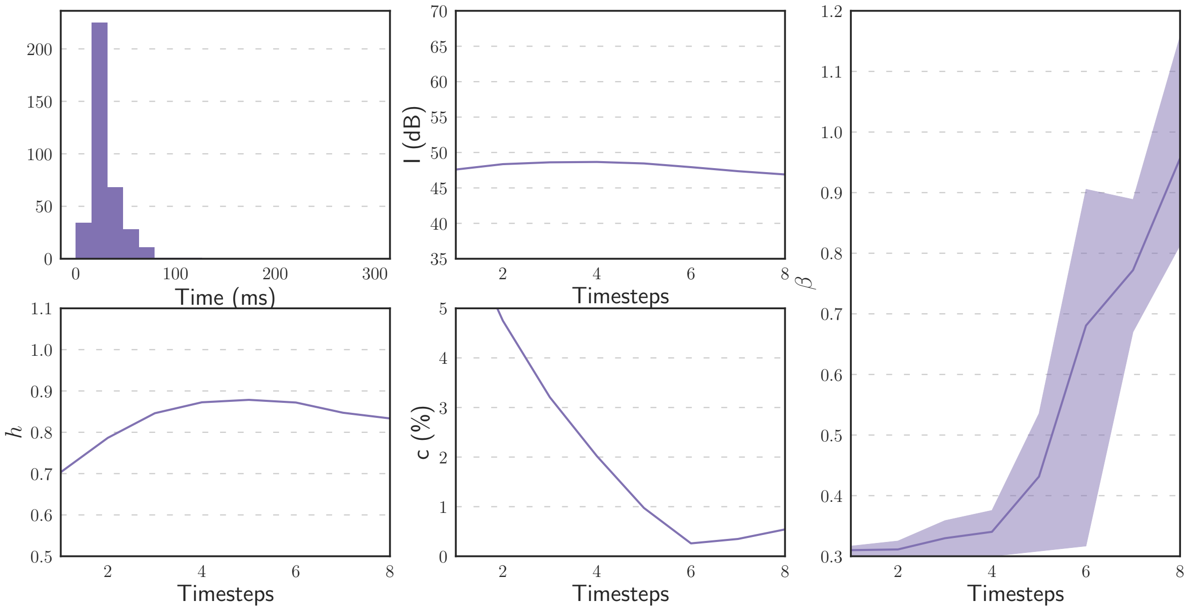

mean duration : 28.4 ms (std : 14.9 ms)

g

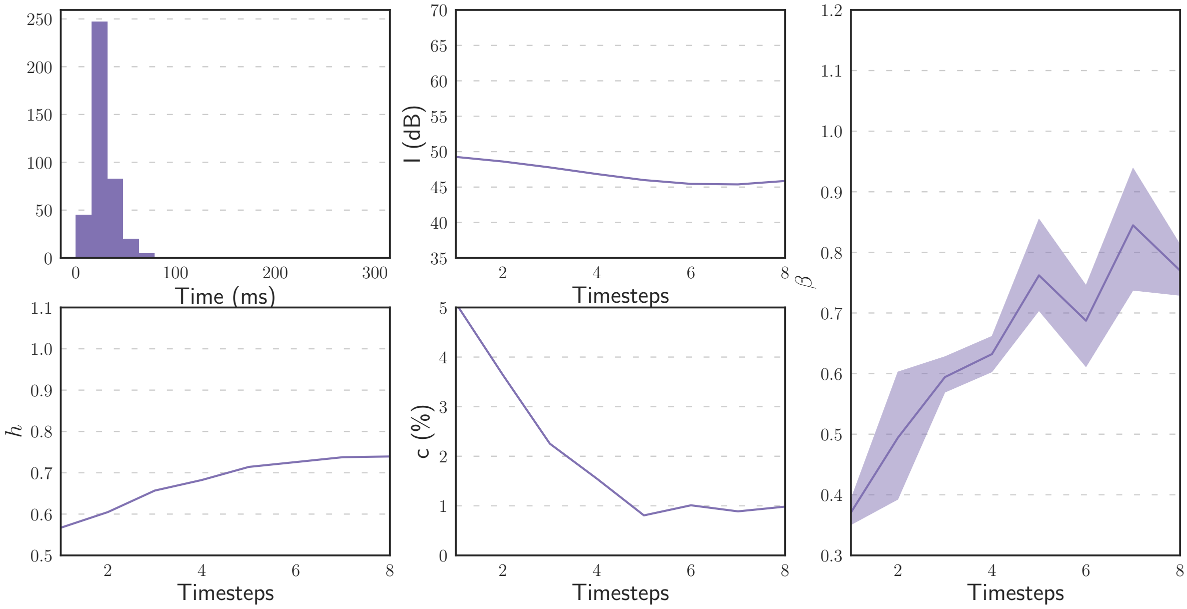

mean duration : 26.5 ms (std : 11.1 ms)

k

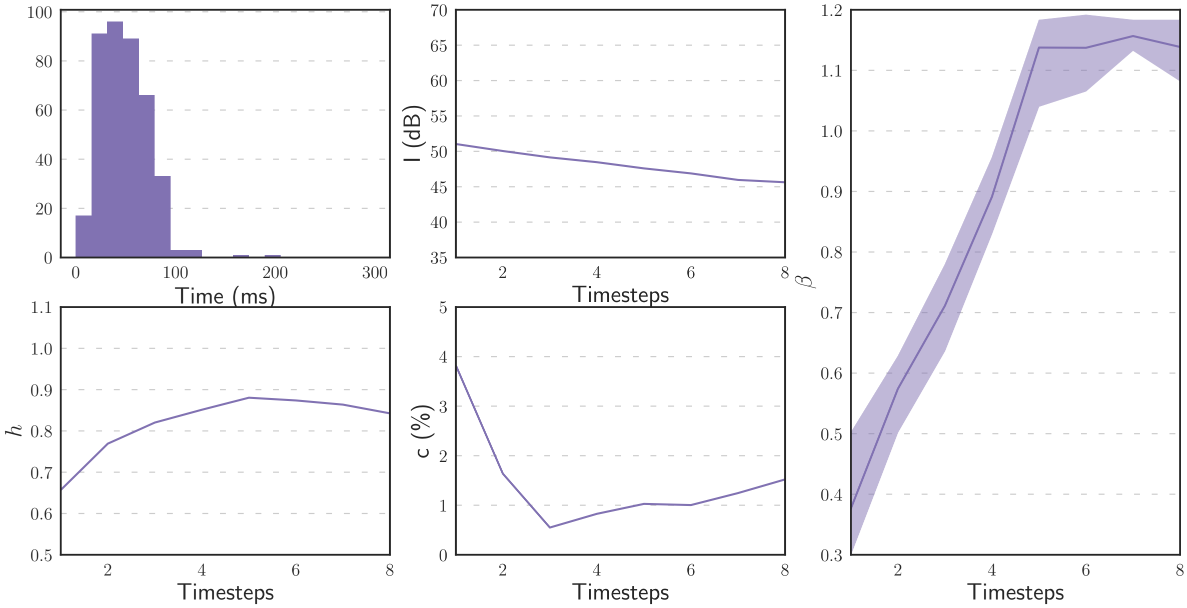

mean duration : 48.9 ms (std : 23.8 ms)

p

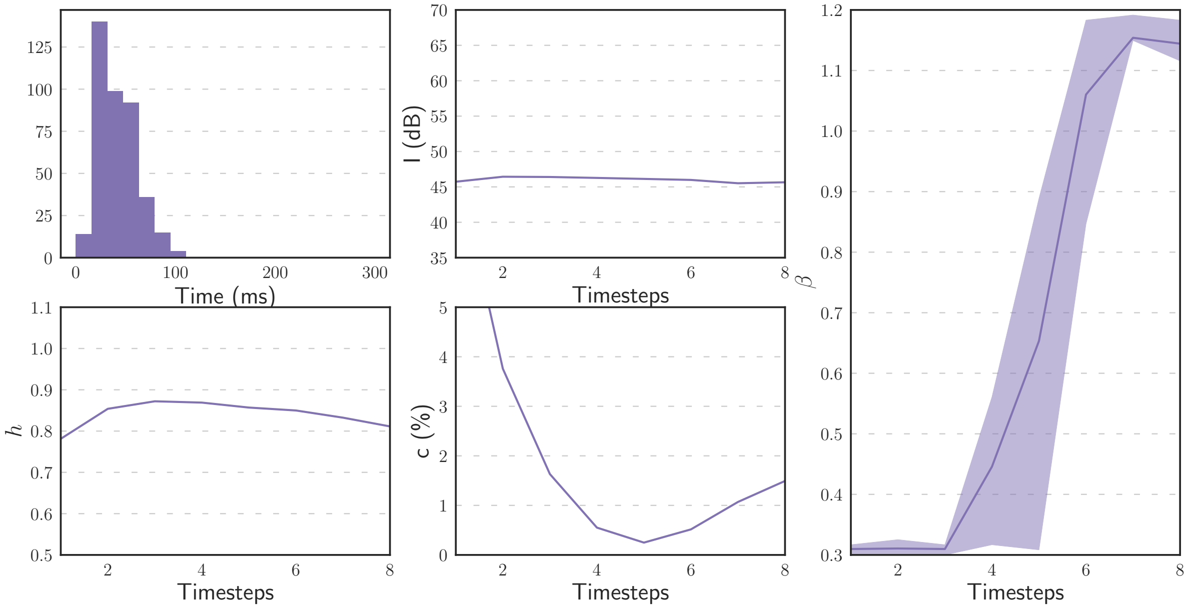

mean duration : 41.9 ms (std : 19.4 ms)

t

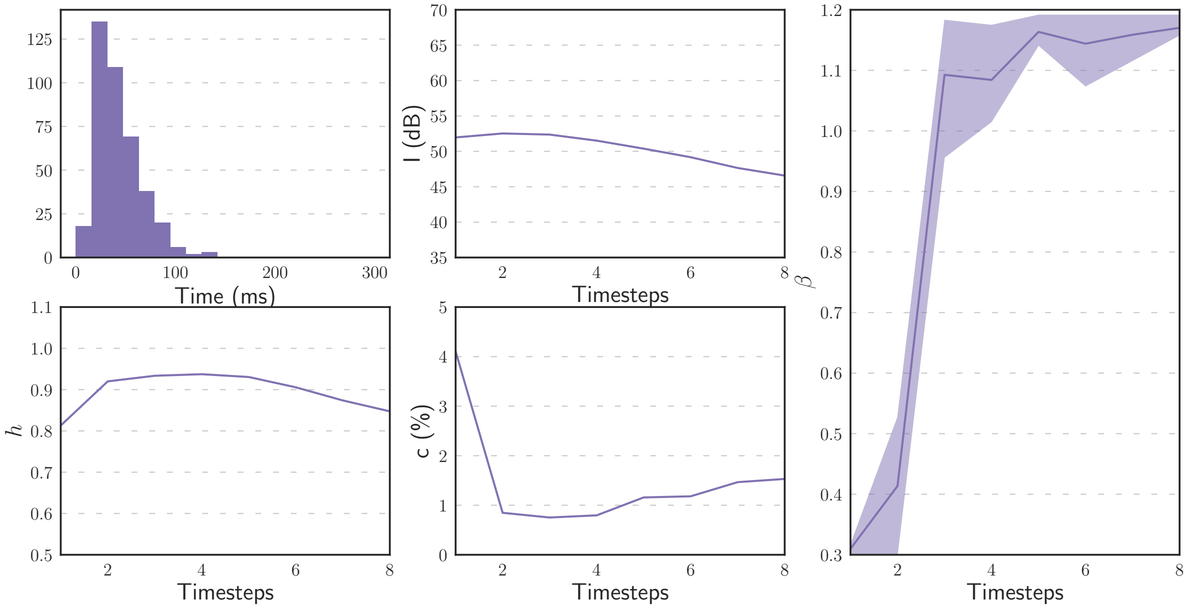

mean duration : 42.9 ms (std : 22.3 ms)

r

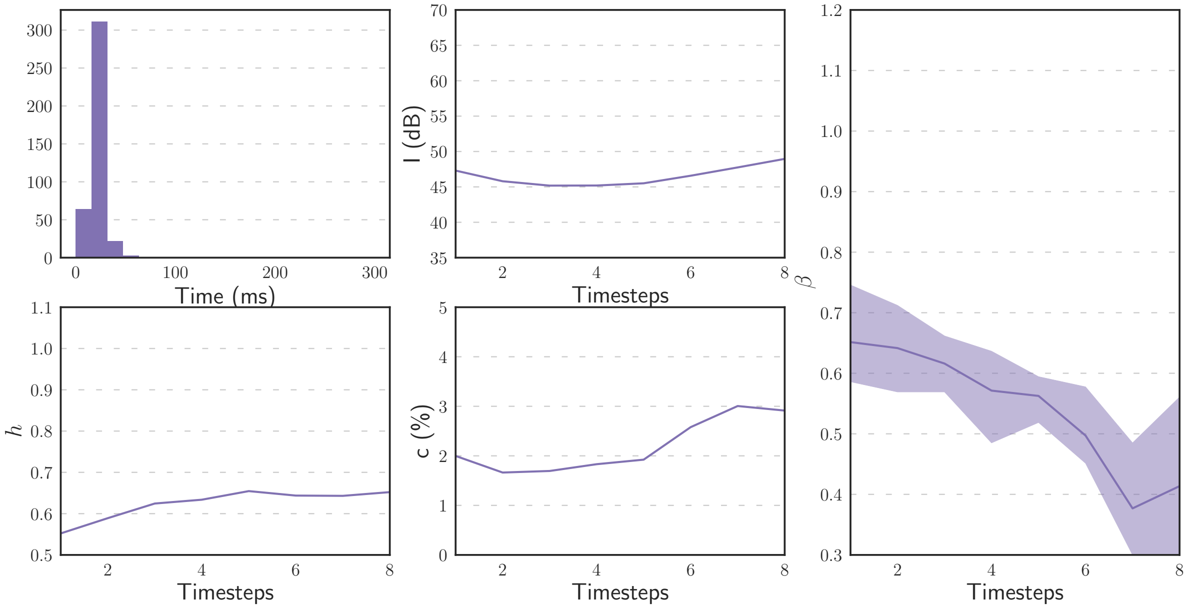

mean duration : 21.1 ms (std : 6.6 ms)

Fricatives

s

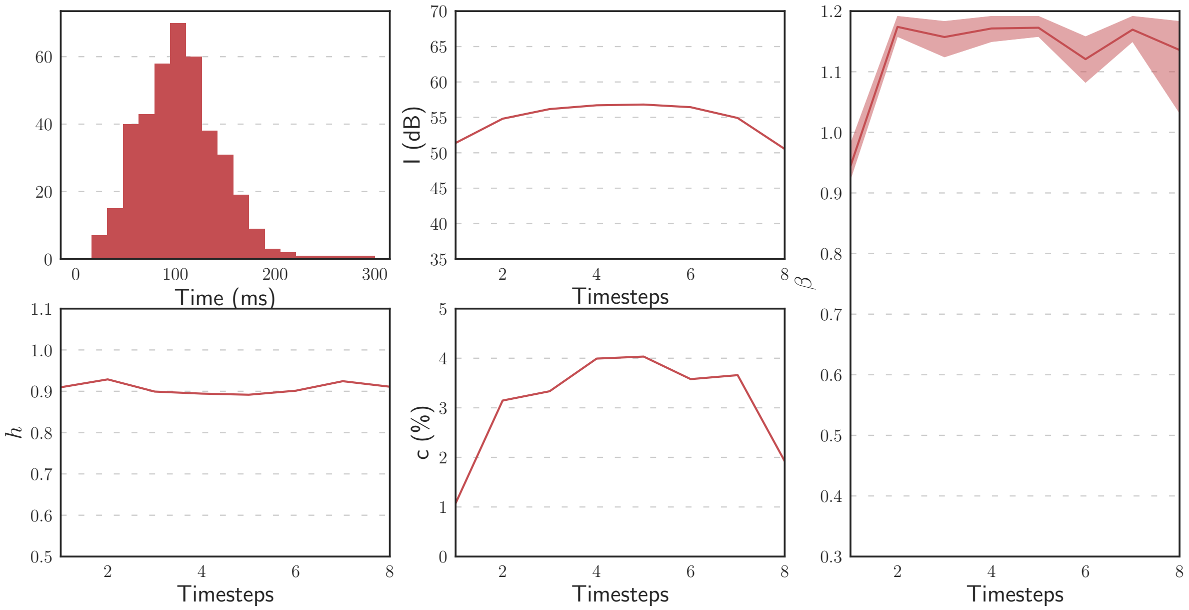

mean duration : 105.8 ms (std : 41.2 ms)

ʃ

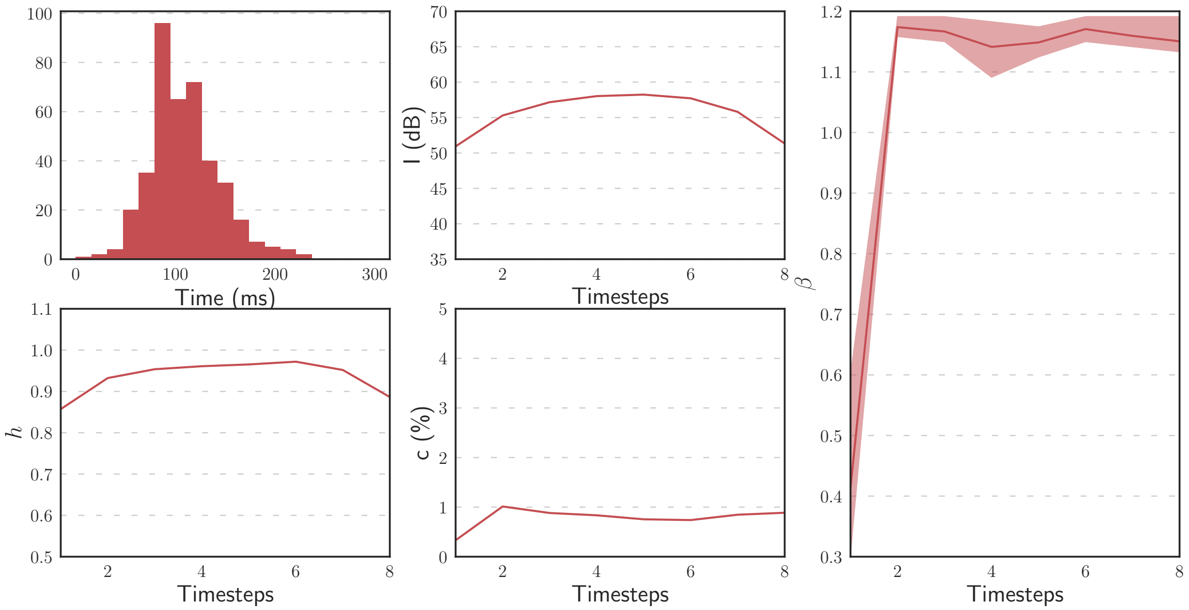

mean duration : 108.8 ms (std : 34.1 ms)

θ

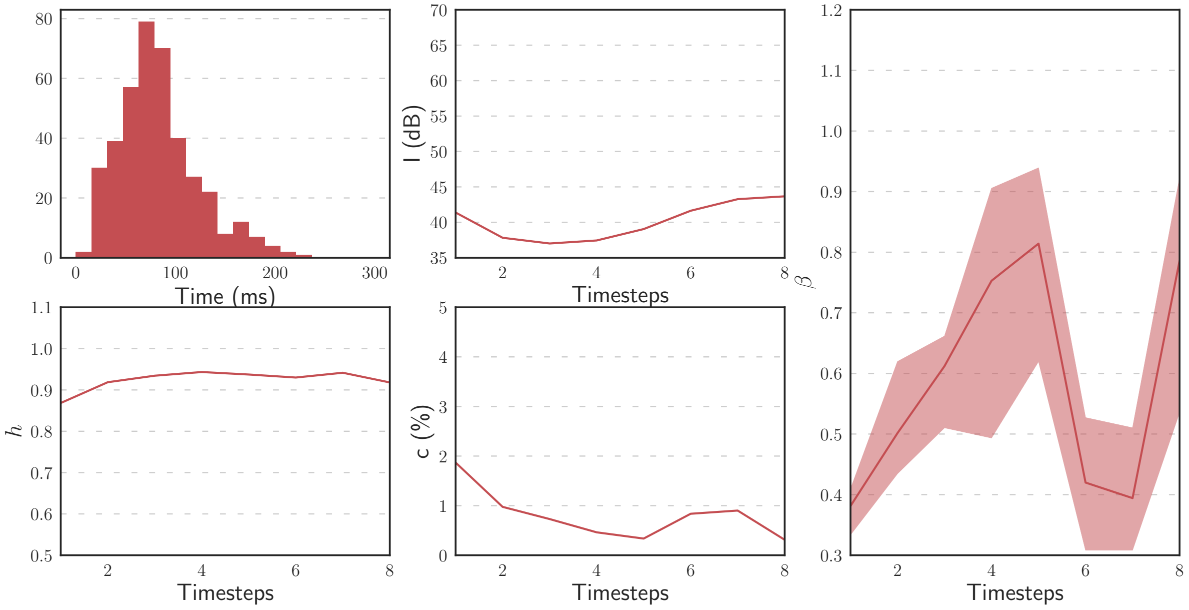

mean duration : 83.4 ms (std : 39.7 ms)

v

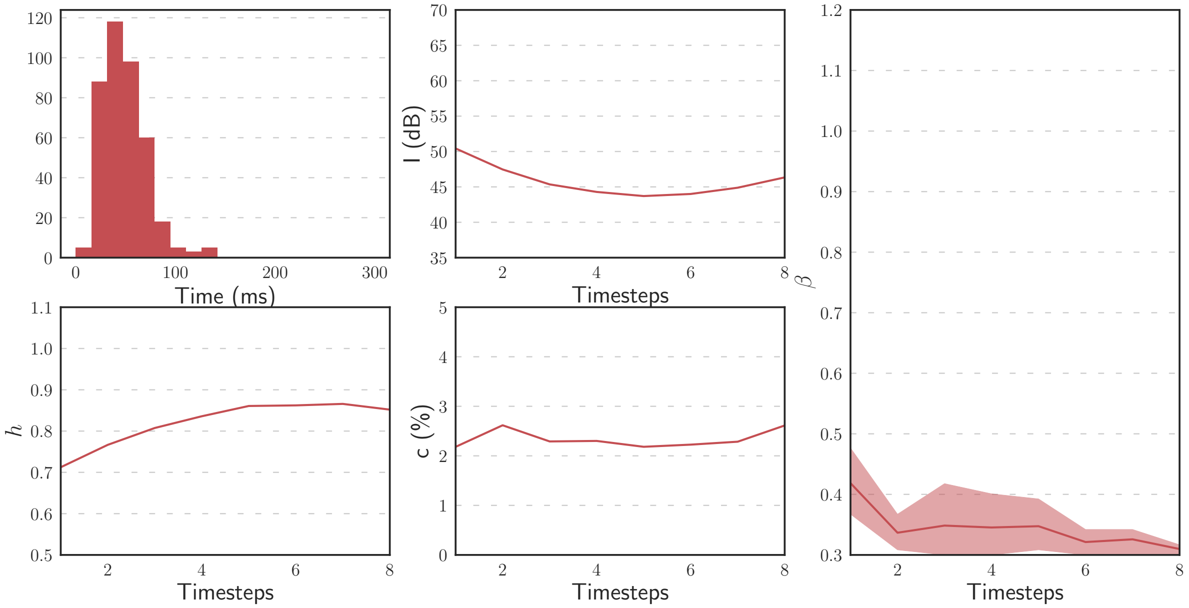

mean duration : 49.1 ms (std : 21.9 ms)

f

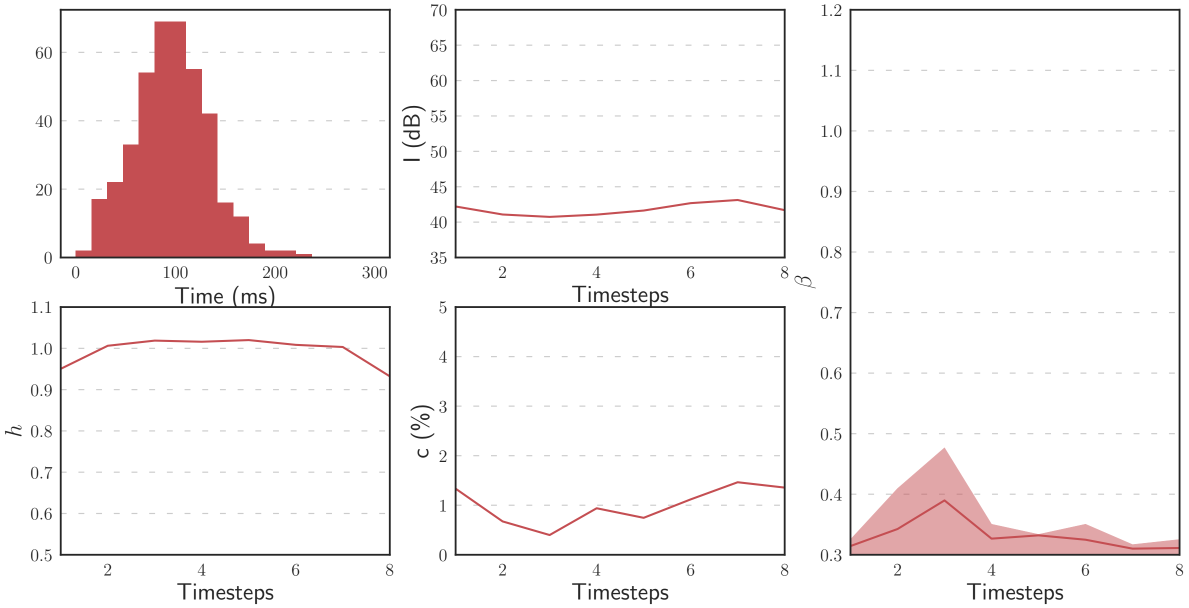

mean duration : 95.8 ms (std : 37.3 ms)

ð

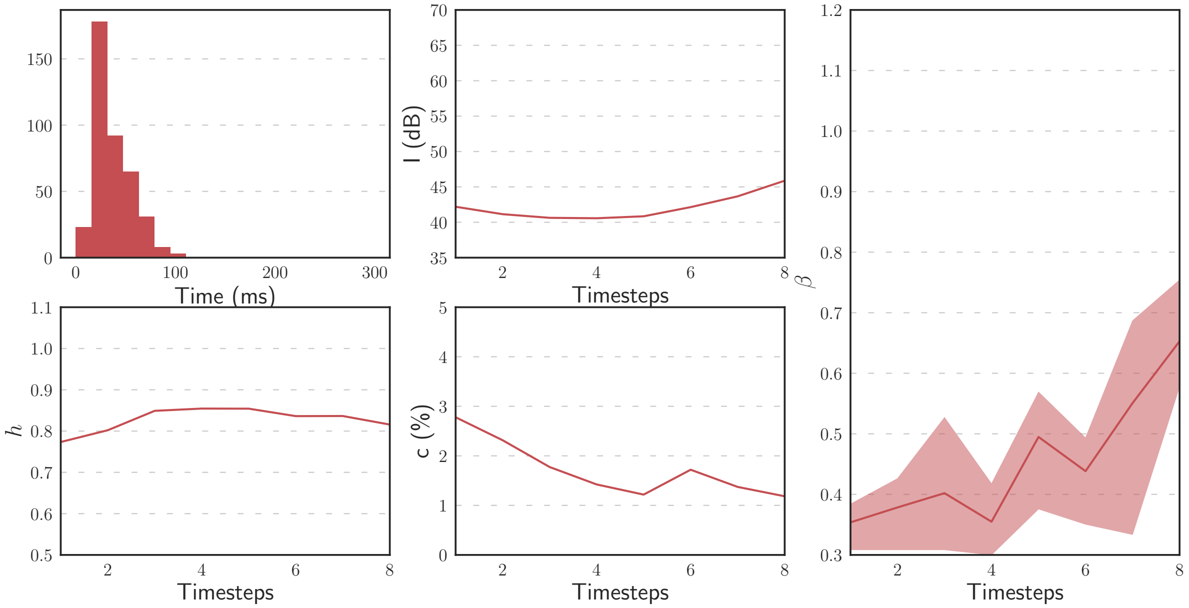

mean duration : 36.4 ms (std : 18.4 ms)

Affricates

tʃ

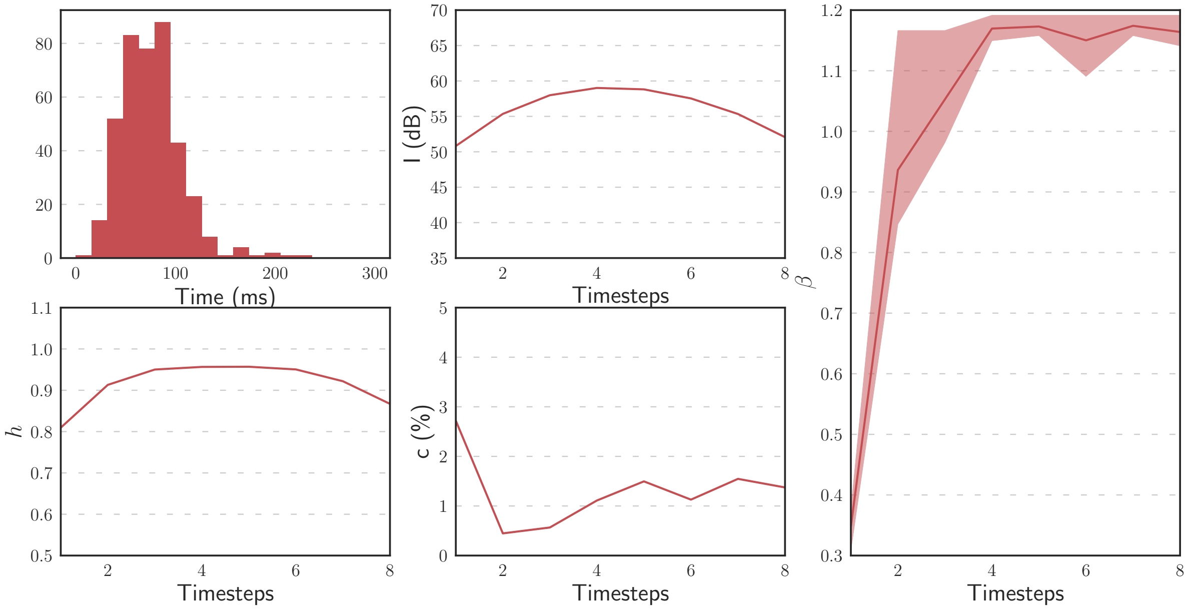

mean duration : 75.1 ms (std : 30.4 ms)

dʒ

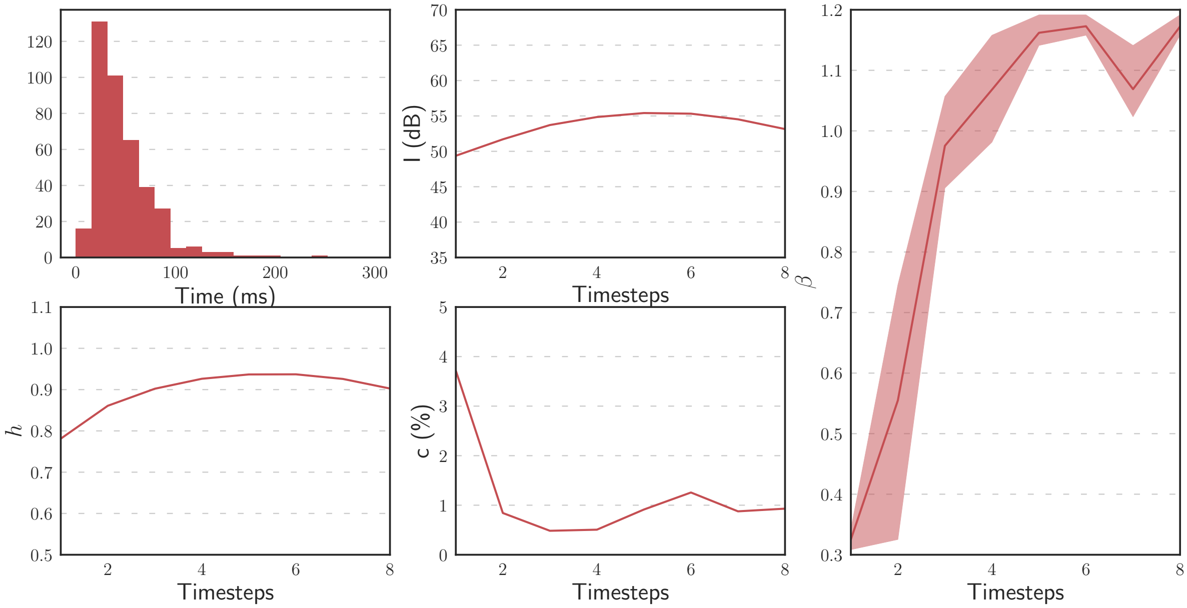

mean duration : 47.0 ms (std : 29.7 ms)
