# Supplementary material for: Fine-grained statistical structure of speech
Source: PLoS One. 2020 Mar 20;15(3):e0230233. doi: 10.1371/journal.pone.0230233 (PMC7083313; doi:10.1371/journal.pone.0230233)
Supplement: S4 Appendix — Complementary analysis and discussion for the estimation of β for single phonemes (vowels and nasals), based on ICA. (PDF) [file pone.0230233.s004.pdf]

Analyses of vowels and nasals using Independent Component Analysis

Objective

This complementary analysis aims to clarify the behavior of Q_{10} as a function of f_c for single phonemes (vowels and nasals). This analysis is based on Independent Component Analysis (ICA). A simultaneous objective is to verify the consistency of the results obtained with the parametric method, described in the main text.

Methods

The experiments repeated the procedure described in Stilp and Lewicki, 2013, for the study of broad phonetic categories. The algorithm and the estimation of the quality factors Q_{10} follow the same path, and are not described in details. The main difference between the two experiments are in the estimation of the Q_{10}/f_c slope, as the lines were constrained to cross a fixed point.

- Inputs: Inputs were concatenated occurrences of the same phoneme, retrieved from the TIMIT database by selecting sentences at random. Each occurrence was windowed by a Tukey window ($\alpha = 0.5$). The files made up of the concatenated samples lasted 1 minute each (1 file corresponding to 1 phoneme).
- Experiments: ICA was conducted 7 times on each file with different initializations. The input vectors were 8-ms slices (dimension: 128 at 16 kHz) extracted at random from the input files. At the beginning of each optimization process, the 128x128 matrix W was a random mixture of time/frequency patterns (impulsions, sinusoids), and noise. The gradient descent was done with approximately 10 000 steps.
- **Estimation of β :** As in the previous work, the β parameter is the regression slope of Q_{10} against center frequency f_c for the filters learned (on a *log-log* scale). The estimation, however, differed here from the previous work as the regression line was constrained to cross the point ($f_0 = 1.0\,\text{kHz}, Q_0 = 2.0$). This choice has been made to be consistent with the parametric representation described in the main text. Therefore, the β parameter was estimated using the following formula :
$$\beta_{ICA} = \frac{\mathbb{E}\left(\log(Q_{10}/Q_0)\log(f_c/f_0)\right)}{\mathbb{E}(\log(f_c/f_0)^2)} \ .$$

The values that are presented in the Results sections are averaged over the 7 experiments.

Results

Figure 1 shows the comparison between the estimates of β using the parametric method (described in the main text) or ICA, on vowels and nasals. Figure 2 and 3 shows the comparison between the two estimates row by row, for vowels and nasals taken apart. These figures also display the bootstrap confidence intervals (parametric method) and the standard deviations on the 7 simulations (ICA). We added the plots of Q_{10} as a function of f_c for most phonemes at the end of the document.

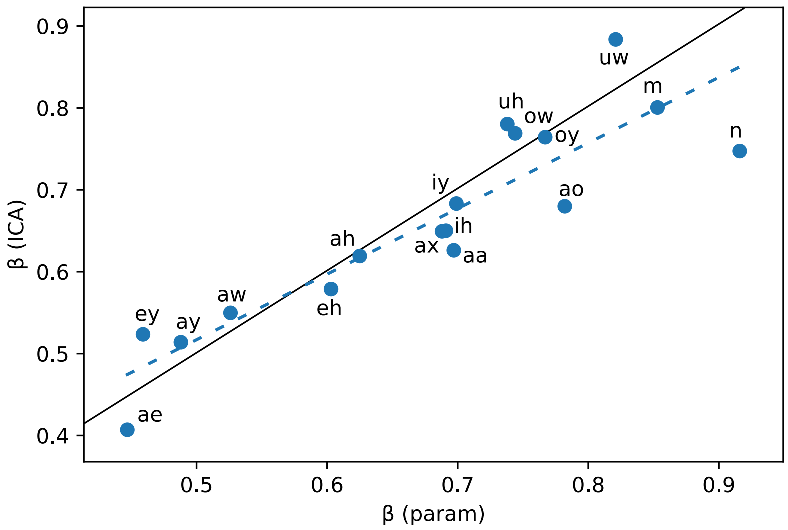

Figure 1: Estimates of β using the parametric method (on the x-axis) and ICA (on the y-axis). The phonemes are indicated with the ARPABET notation. The dark line is the diagonal and the blue line is the linear regression connecting the points (slope:0.80, intercept:0.12). Standard deviation from the diagonal : 0.058, from the linear regression: 0.052. The correlation coefficient is r=0.90.

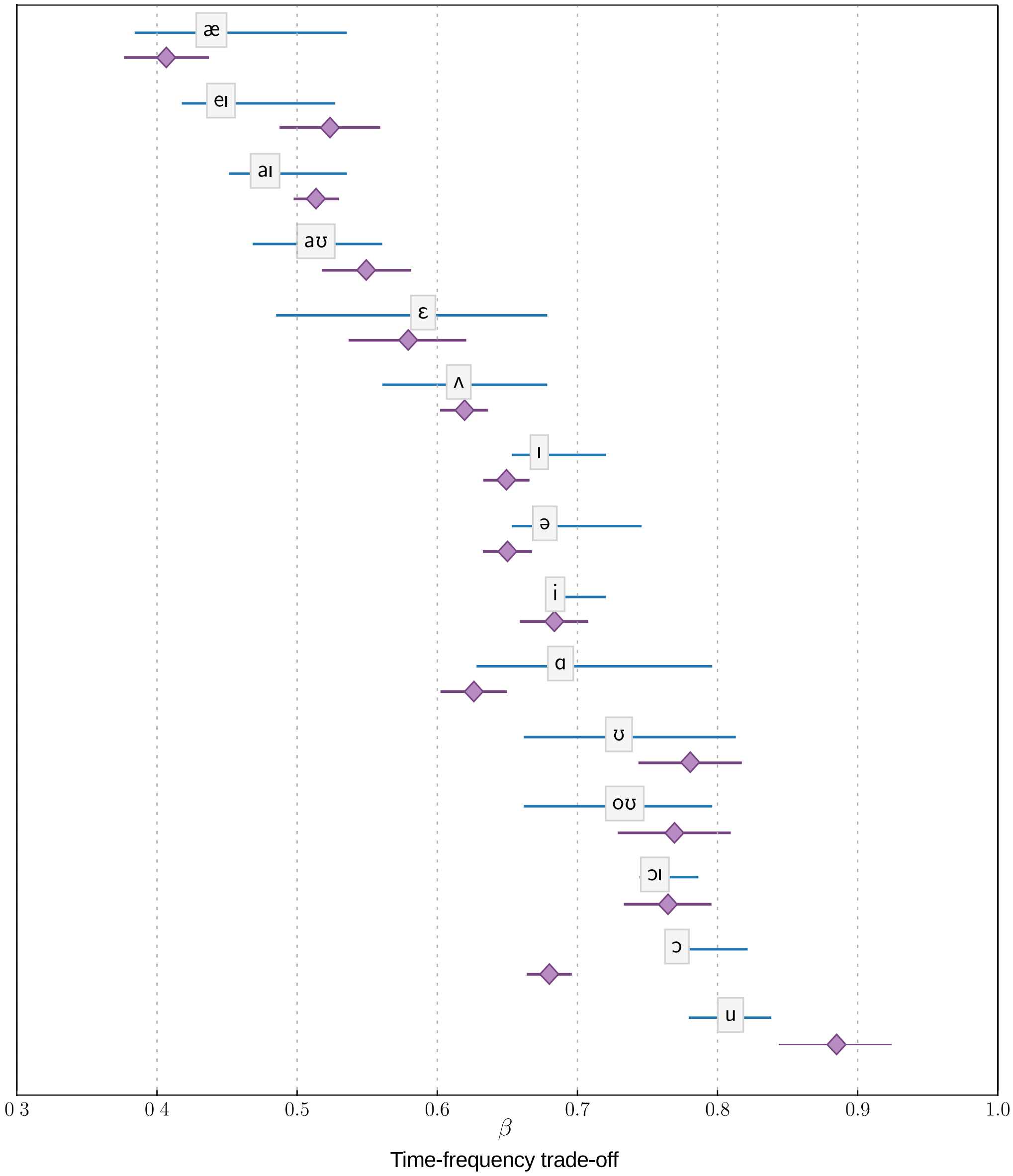

Figure 2: Estimates of β using the parametric method (labels) and ICA (diamonds) for vowels. The diagram shows the 70% bootstrap confidence intervals (parametric method) and +/- standard deviations computed on 7 simulations (ICA).

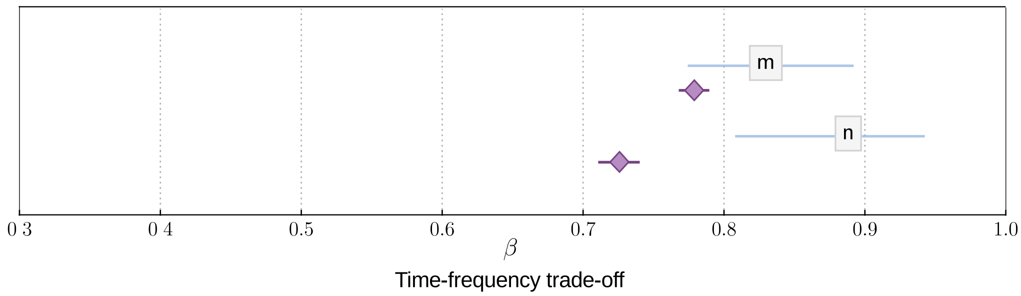

Figure 3: Estimates of β using the parametric method (labels) and ICA (diamonds) for nasals.

Discussion

The comparison between ICA and the parametric method shows the overall consistency of the β estimates and confirm the main conclusions on the statistical structure of vowels and nasals, detailed in the main text. The largest differences are found for the phonemes [n], [ɔ] (gap greater than 0.1), followed by [ɑ], [ei], [u][1]. All the other phonemes exhibit differences of less than 0.6.

The figures of the quality factors as a function of frequency, provided in the section below, offer additional insights. The main differences in the quality factors between vowels are shown to be in the region 1–5 kHz. In particular, mid-open vowels ([ɛ], [ʌ]), diphthongs (e.g. [ai]), but especially the sound [æ], can produce very low quality factors below 3 kHz. This fact accounts for a significant difference in behavior between this analysis and Stilp and Lewicki's analysis, potentially leading to opposing observations. The low quality factors at medium frequencies make the regression slope steeper in the unconstrained setting – as it is the case in Stilp and Lewicki's work. For instance, the diphthongs [ai] or [ei] are associated with high β values if the regression is unconstrained (~ 1), but with much lower values if constrained (~ 0.5). This fact also explains why the β values found in this analysis are generally lower than those previously reported for vowels and nasals. The figures reveal several local behaviors. The Q_{10} factors are higher in regions where formants are found, especially between 1 and 2 kHz. For example, the *schwa* [ə] and the related sound [ʌ] both present a "bump" at $f_c = 1.5\,\text{kHz}$. This effect is also visible when comparing the plots between back vowels (e.g. [ɔ]), which have a low second formant ($\sim 1\,\text{kHz}$), and front vowels (e.g [i], [ɪ]), which have higher second formant ($> 2\,\text{kHz}$). Another local behavior is the rise of Q_{10} near an antiresonance, particularly visible for the sound [m], close to $f_c = 3\,\text{kHz}$ (second antiresonance of the mouth cavity). This behavior, however, is not systematic (not apparent on [n]).

Additional figures

In this section we provide examples of plots of Q_{10} as a function of f_c. The selected examples were the ones that provided the β estimate closest to the average value (for the same phoneme).

The figures show the regression lines both in the constrained and the unconstrained (standard regression) cases.

[1] In the other experiments based on different weightings strategies (appendix S1), the values for [ɔ], [ɑ] were consistent with ICA. It is possible that the chosen settings for the main figure (Strategy C) is not the most appropriate for the low back vowels.

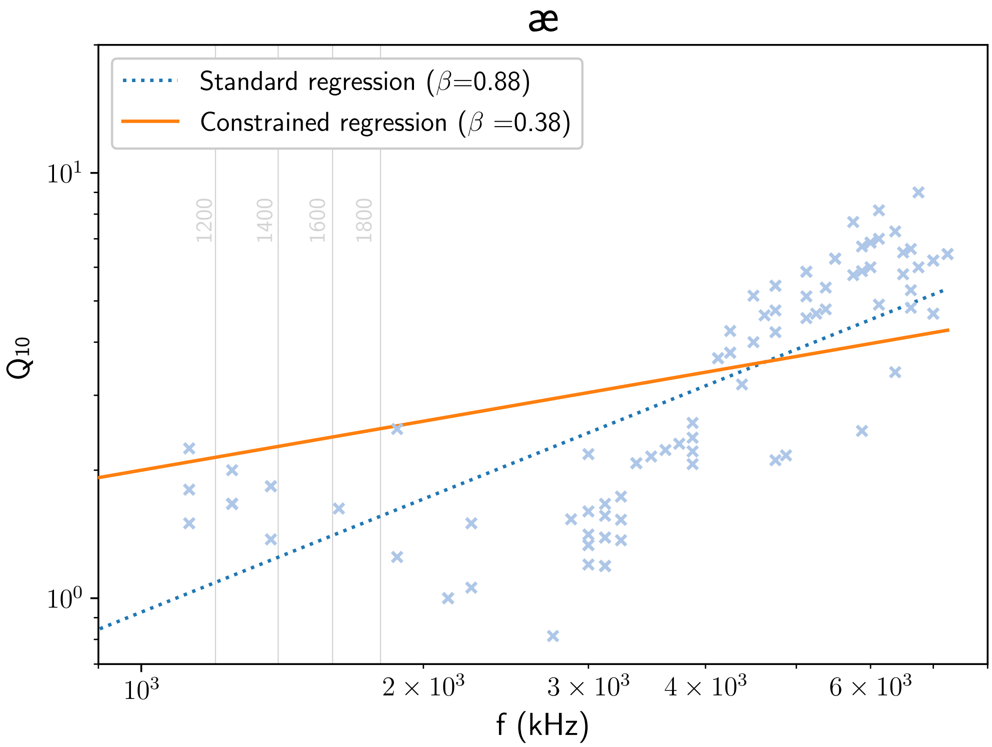
æ
Standard regression (β=0.88)
Constrained regression (β =0.38)
1200
1400
1600
1800
Q10
f (kHz)

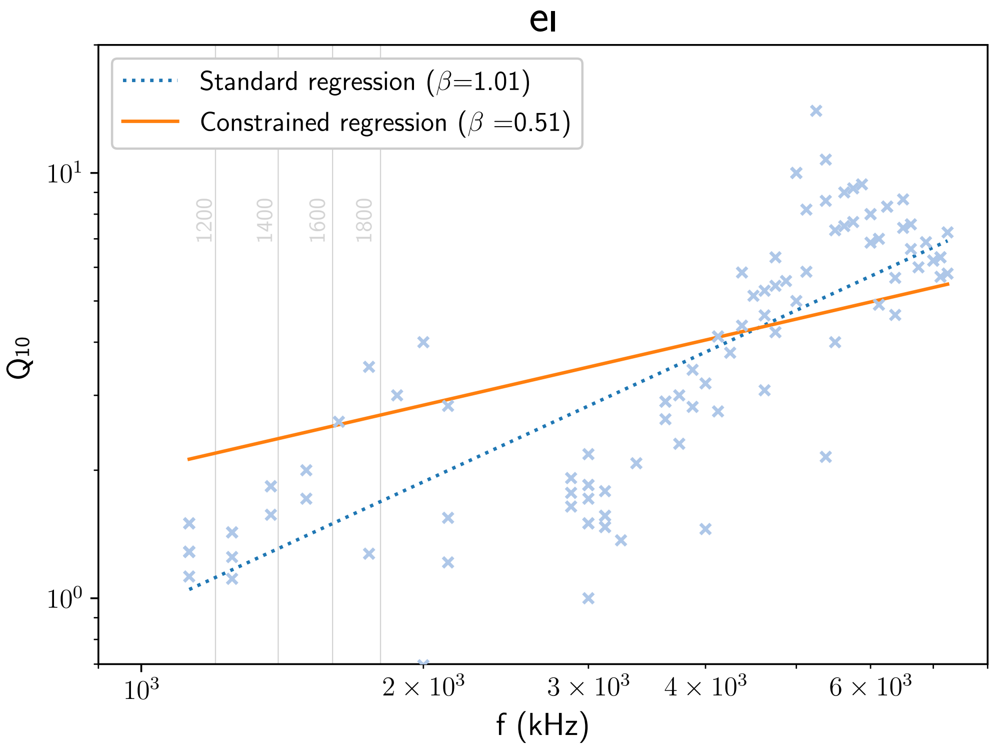
eɪ
Standard regression (β=1.01)
Constrained regression (β =0.51)
1200
1400
1600
1800
Q10
f (kHz)

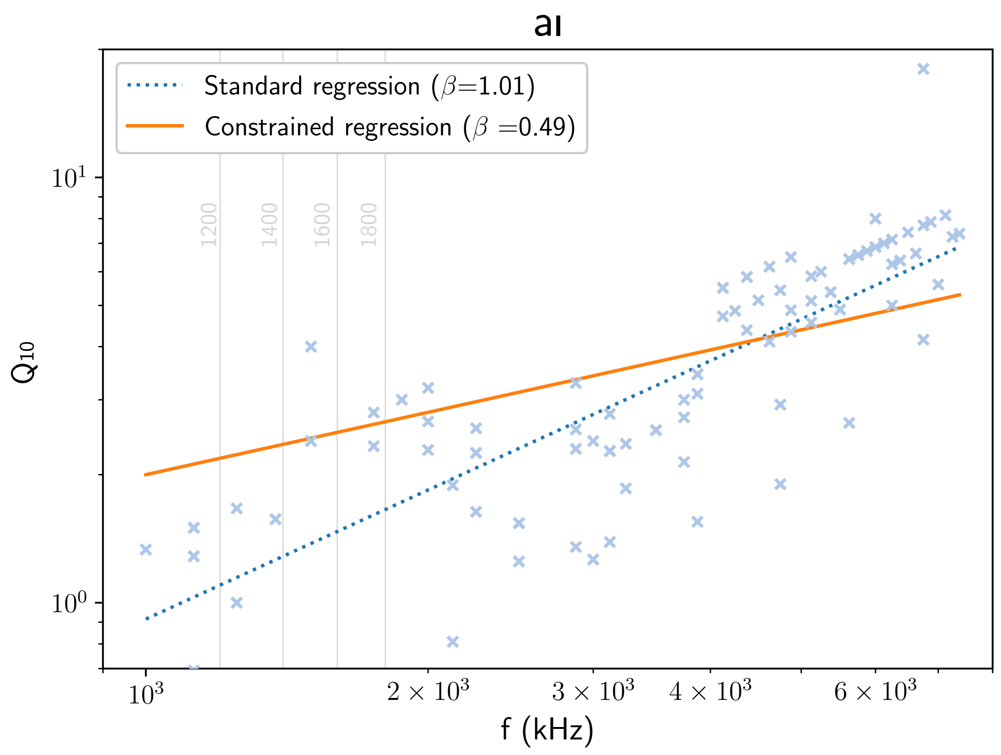
aɪ
Standard regression (β=1.01)
Constrained regression (β =0.49)
1200
1400
1600
1800
10^1
10^0
Q_{10}
10^3
2×10^3
3×10^3
4×10^3
6×10^3
f (kHz)

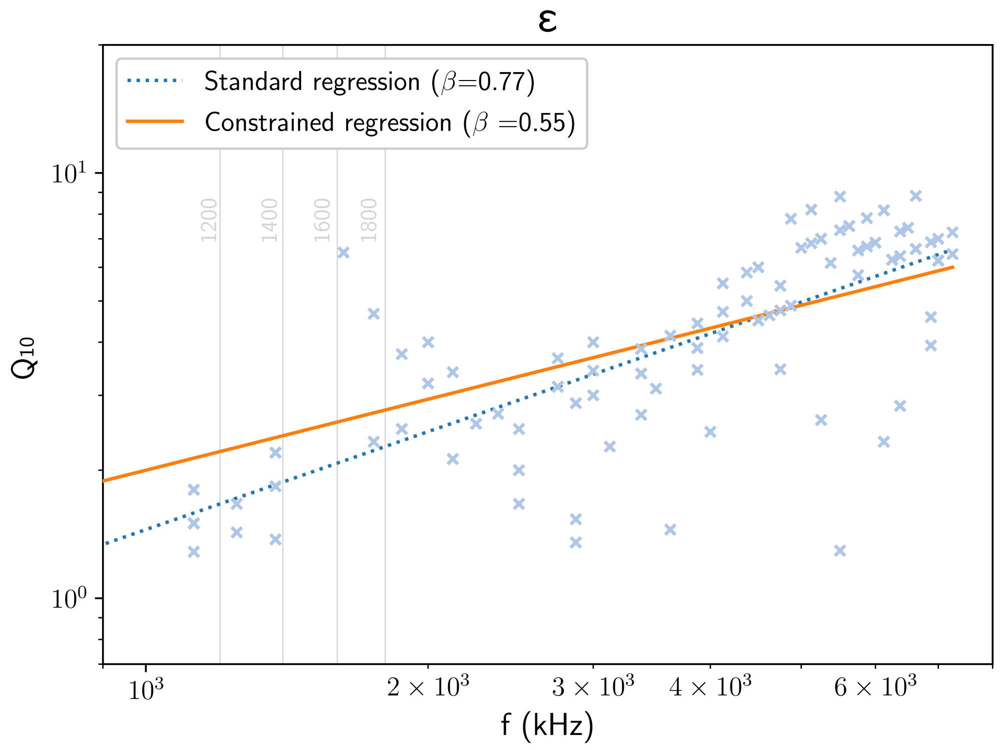
ɛ
Standard regression (β=0.77)
Constrained regression (β =0.55)
1200
1400
1600
1800
10^1
10^0
Q_{10}
10^3
2×10^3
3×10^3
4×10^3
6×10^3
f (kHz)

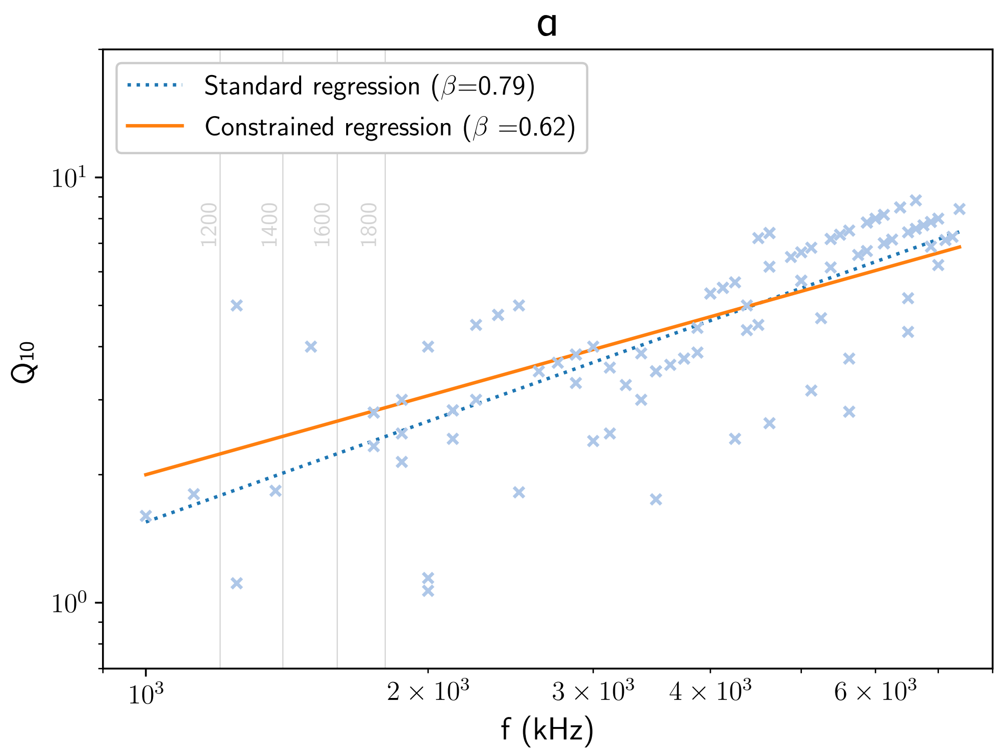

ɑ
Standard regression (β=0.79)
Constrained regression (β =0.62)
1200
1400
1600
1800
10^1
10^0
Q_{10}
10^3
2×10^3
3×10^3
4×10^3
6×10^3
f (kHz)

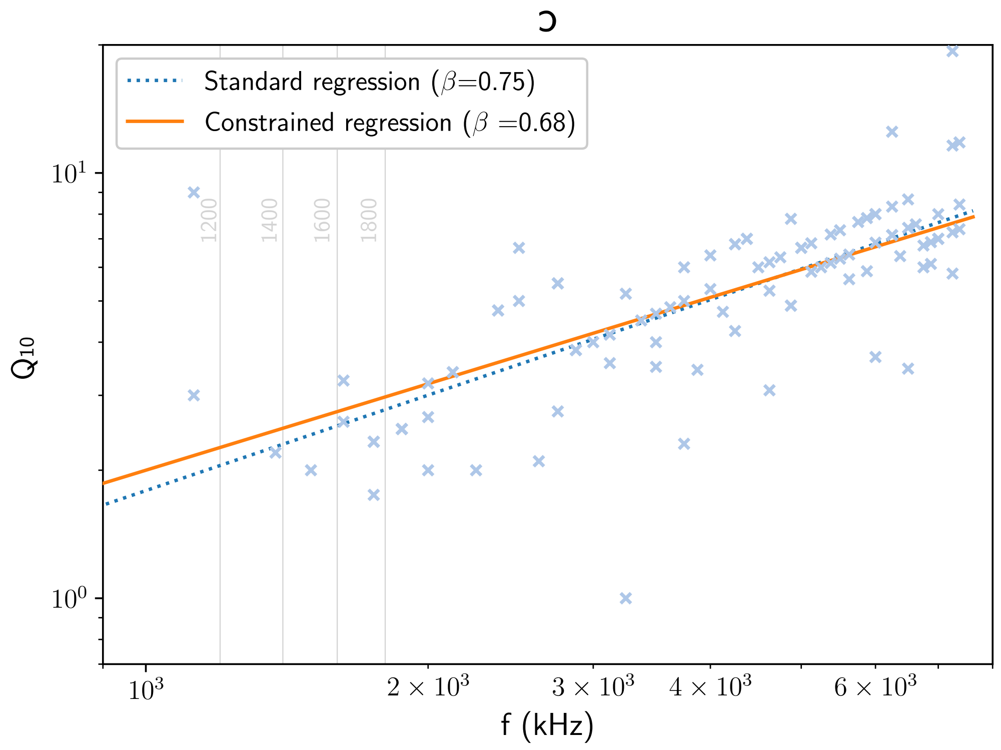

ɔ
Standard regression (β=0.75)
Constrained regression (β =0.68)
1200
1400
1600
1800
10^1
10^0
Q_{10}
10^3
2×10^3
3×10^3
4×10^3
6×10^3
f (kHz)

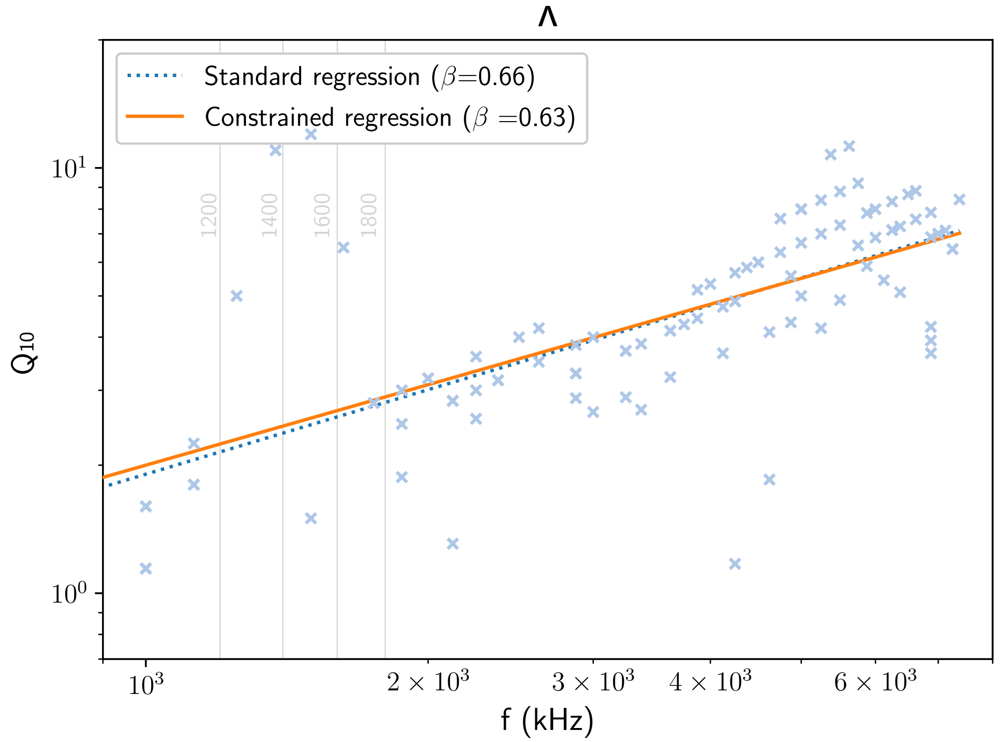
ʌ
Standard regression (β=0.66)
Constrained regression (β =0.63)
1200
1400
1600
1800
Q10
f (kHz)

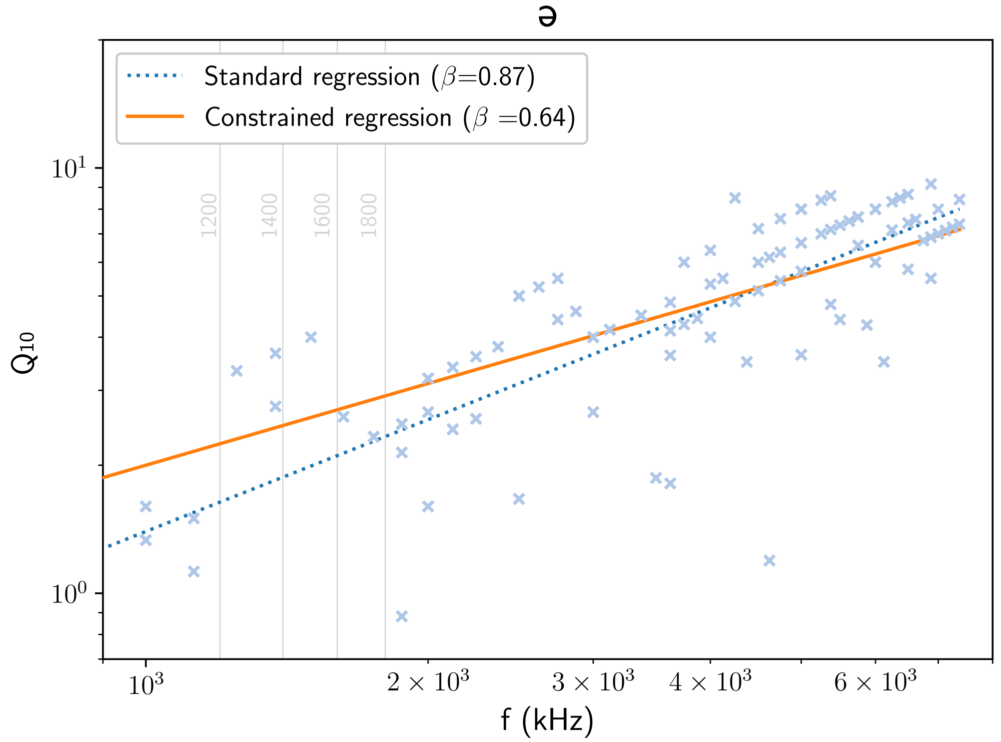
ə
Standard regression (β=0.87)
Constrained regression (β =0.64)
1200
1400
1600
1800
Q10
f (kHz)

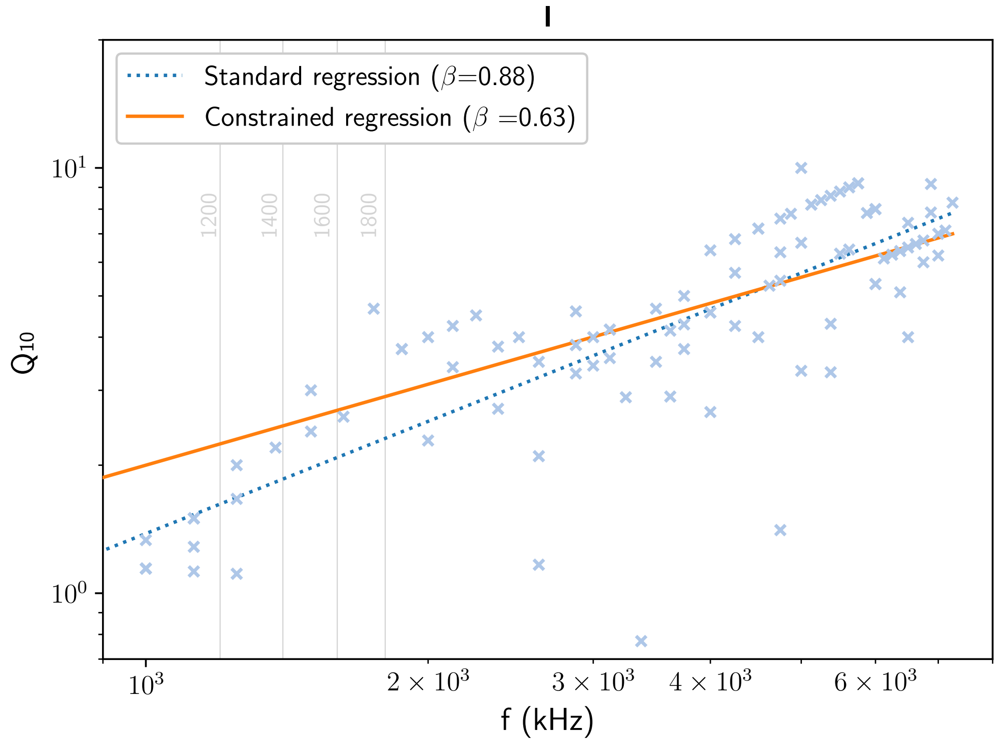
I
Standard regression (β=0.88)
Constrained regression (β =0.63)
10^1
10^0
Q10
1200
1400
1600
1800
10^3
2×10^3
3×10^3
4×10^3
6×10^3
f (kHz)

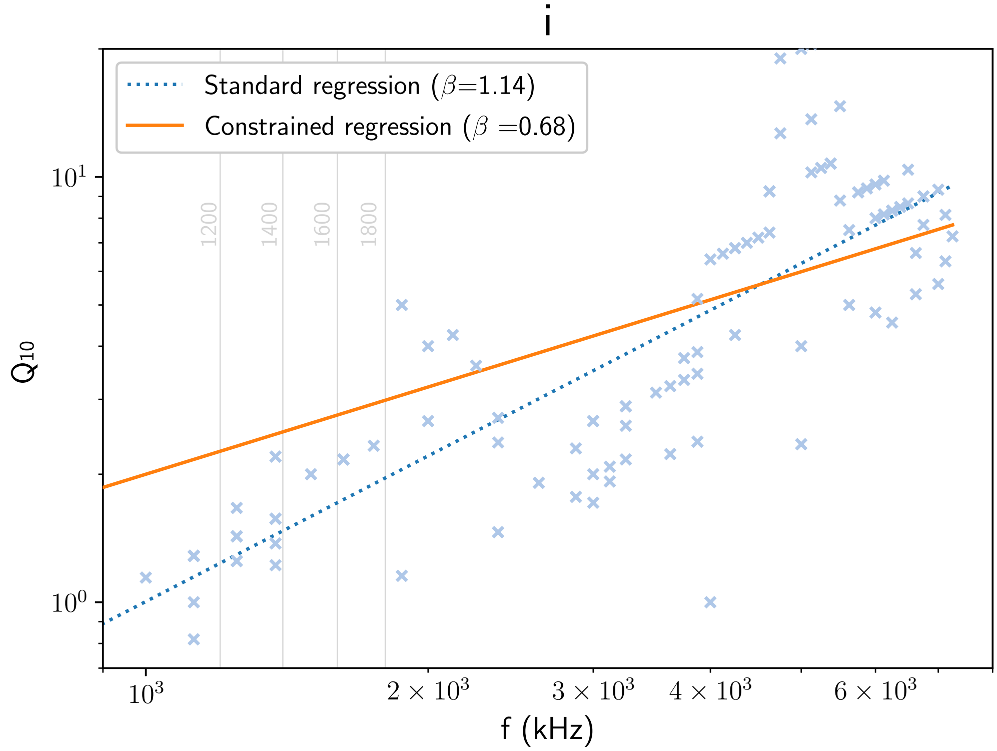
i
Standard regression (β=1.14)
Constrained regression (β =0.68)
10^1
10^0
Q10
1200
1400
1600
1800
10^3
2×10^3
3×10^3
4×10^3
6×10^3
f (kHz)

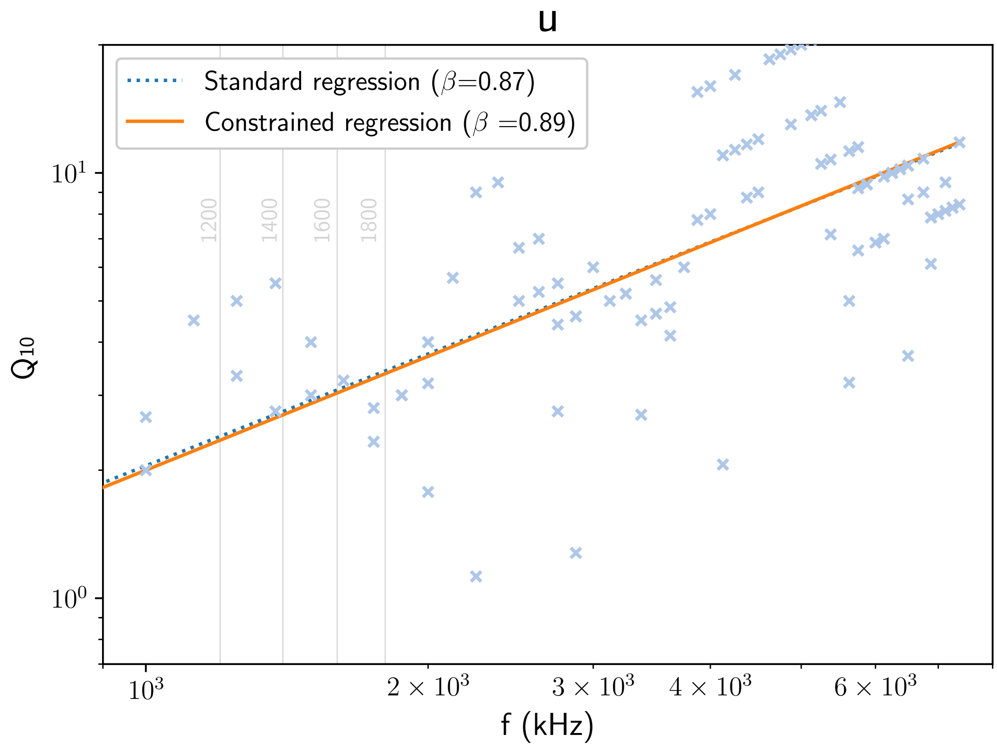
ɯ
Standard regression (β=0.87)
Constrained regression (β =0.89)
1200
1400
1600
1800
Q_{10}
10^1
10^0
10^3
2×10^3
3×10^3
4×10^3
6×10^3
f (kHz)

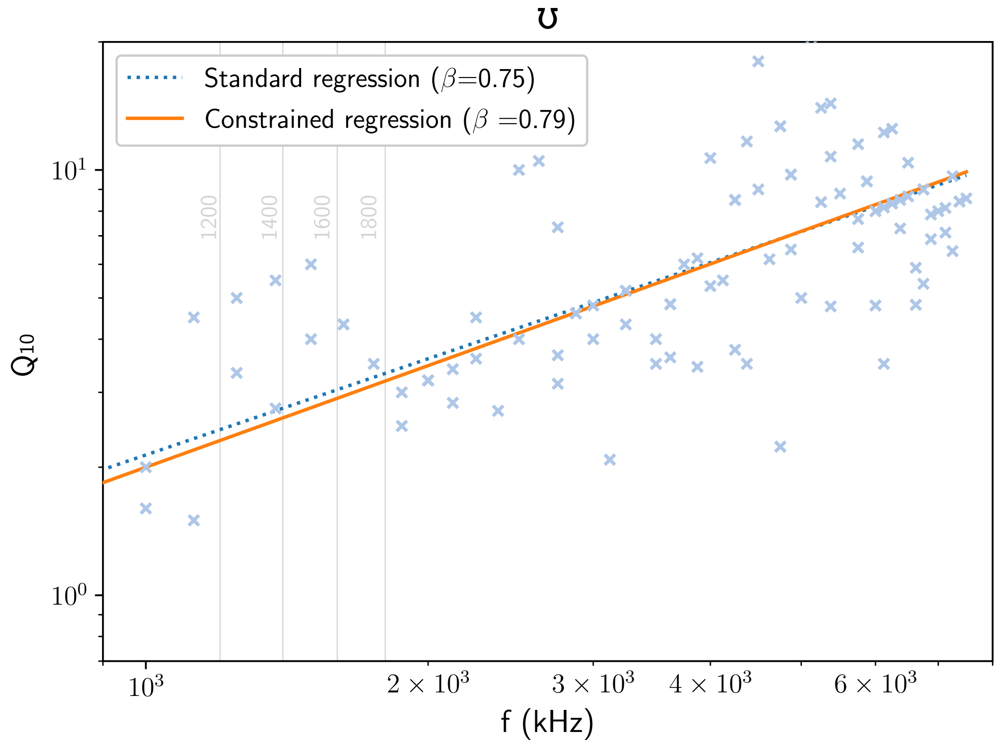
ʊ
Standard regression (β=0.75)
Constrained regression (β =0.79)
1200
1400
1600
1800
Q_{10}
10^1
10^0
10^3
2×10^3
3×10^3
4×10^3
6×10^3
f (kHz)

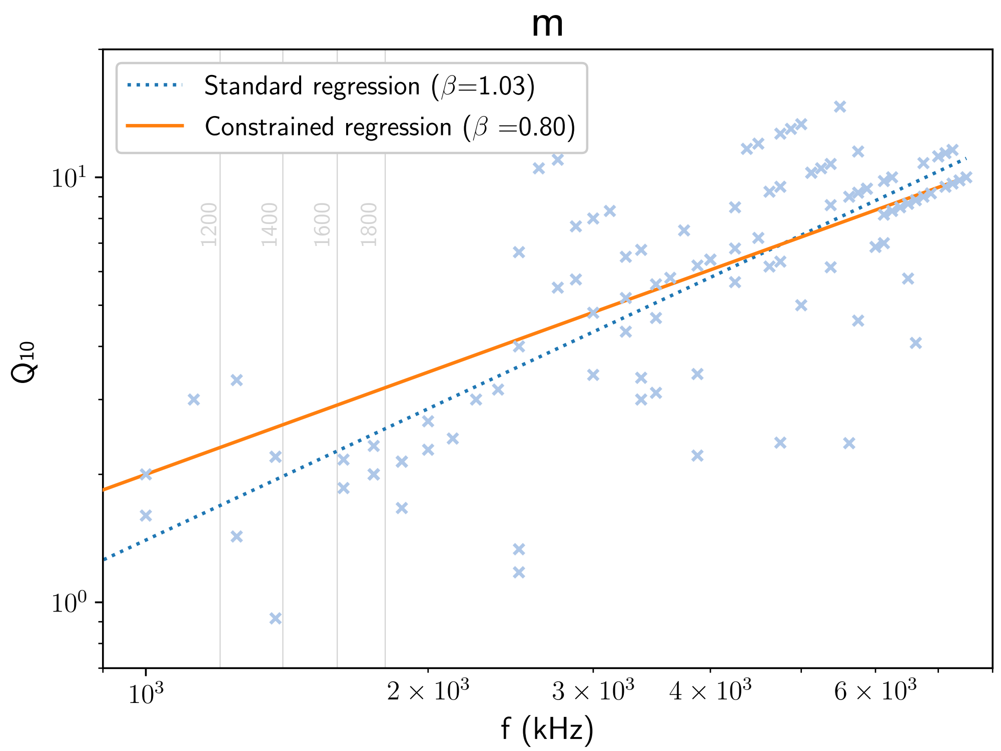

m
Standard regression (β=1.03)
Constrained regression (β =0.80)
1200
1400
1600
1800
10^1
10^0
Q_{10}
10^3
2×10^3
3×10^3
4×10^3
6×10^3
f (kHz)

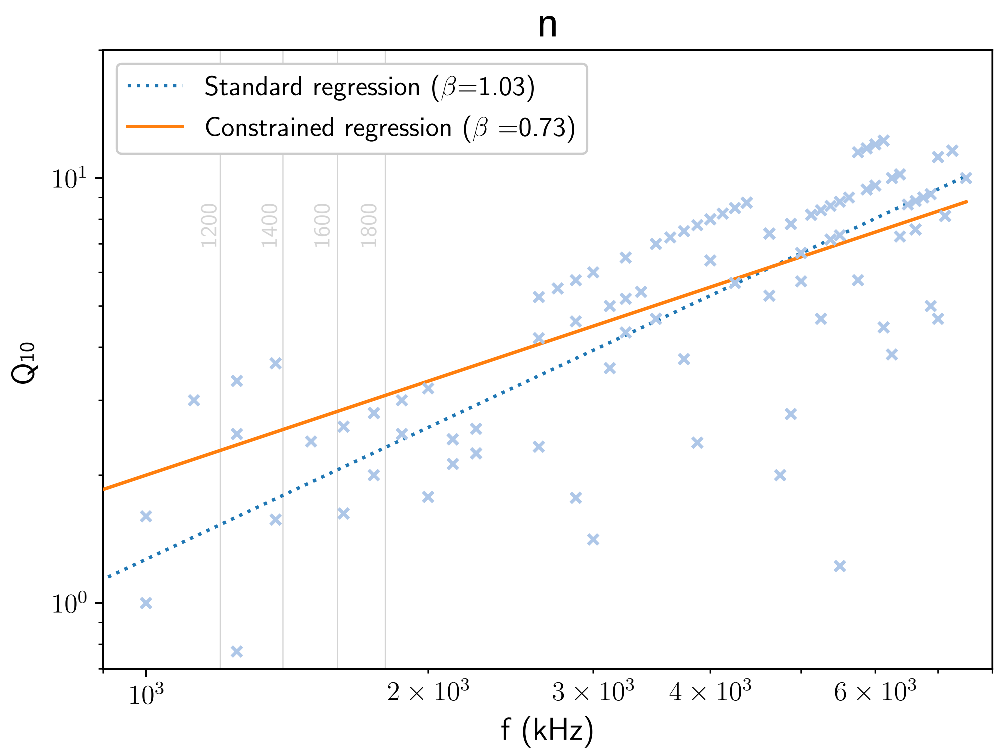

n
Standard regression (β=1.03)
Constrained regression (β =0.73)
1200
1400
1600
1800
10^1
10^0
Q_{10}
10^3
2×10^3
3×10^3
4×10^3
6×10^3
f (kHz)
