# Supplementary material for: Fine-grained statistical structure of speech
Source: PLoS One. 2020 Mar 20;15(3):e0230233. doi: 10.1371/journal.pone.0230233 (PMC7083313; doi:10.1371/journal.pone.0230233)
Supplement: S1 Fig — (PDF) [file pone.0230233.s006.pdf]

β as a function of intensity level for synthetic vowel-like sounds

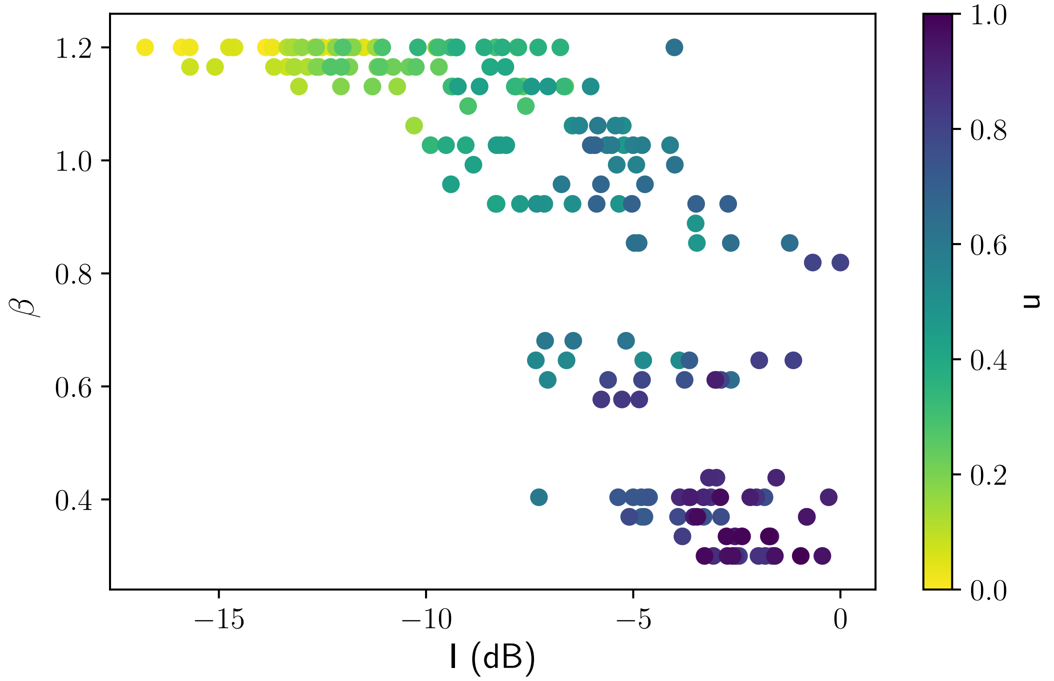

Figure: Scatter plot of simulated samples on the (I, β) plane: exponent β against intensity I in dB (ref:max). Each point is a sample of Simulation 2 on synthesized vowels. The parameter u controls linearly the aperture of the cylindrical waveguide, from $r = 0.2cm$ $(u = 0)$ to $r = 1.3cm$ $(u = 1)$.
